# Identification of Key Signaling Pathways Orchestrating Substrate Topography Directed Osteogenic Differentiation Through High-Throughput siRNA Screening

**DOI:** 10.1038/s41598-018-37554-y

**Published:** 2019-01-30

**Authors:** Tugba Ozdemir, Daniel T. Bowers, Xiang Zhan, Debashis Ghosh, Justin L. Brown

**Affiliations:** 1 Department of Genetics and Bioengineering, Tokat Gaziosmanpasa University, Tokat, Turkey; 2 Department of Biomedical Engineering, The Pennsylvania State University, University Park, State College, PA USA; 3 Department of Public Health Sciences, Penn State College of Medicine, Hershey, PA USA; 4 Department of Biostatistics and Informatics, Colorado School of Public Health, Aurora, CO USA

## Abstract

Fibrous scaffolds are used for bone tissue engineering purposes with great success across a variety of polymers with different physical and chemical properties. It is now evident that the correct degree of curvature promotes increased cytoskeletal tension on osteoprogenitors leading to osteogenic differentiation. However, the mechanotransductive pathways involved in this phenomenon are not fully understood. To achieve a reproducible and specific cellular response, an increased mechanistic understanding of the molecular mechanisms driving the fibrous scaffold mediated bone regeneration must be understood. High throughput siRNA mediated screening technology has been utilized for dissecting molecular targets that are important in certain cellular phenotypes. In this study, we used siRNA mediated gene silencing to understand the osteogenic differentiation observed on fibrous scaffolds. A high-throughput siRNA screen was conducted using a library collection of 863 genes including important human kinase and phosphatase targets on pre-osteoblast SaOS-2 cells. The cells were grown on electrospun poly(methyl methacrylate) (PMMA) scaffolds with a diameter of 0.938 ± 0.304 µm and a flat surface control. The osteogenic transcription factor RUNX2 was quantified with an in-cell western (ICW) assay for the primary screen and significant targets were selected via two sample t-test. After selecting the significant targets, a secondary screen was performed to identify osteoinductive markers that also effect cell shape on fibrous topography. Finally, we report the most physiologically relevant molecular signaling mechanisms that are involved in growth factor free, fibrous topography mediated osteoinduction. We identified GTPases, membrane channel proteins, and microtubule associated targets that promote an osteoinductive cell shape on fibrous scaffolds.

## Introduction

Synthetic bone constructs can function as alternatives to autograft, allograft and xenograft tissue sources. These natural sources of tissue have a complex mixture of signaling molecules and extracellular matrix topography that is osteoinductive. Therefore, in order for synthetic substitutes to be effective, a mechanistic understanding of the processes by which the soluble and intrinsic shape environmental cues are sensed by cells is required. Mesenchymal Stem Cells (MSCs), identified from a bone marrow aspirate by their ability to: adhere to a surface, express a panel of markers (CD105^+^, CD73^+^, CD90^+^, CD34^−^, CD45^−^, CD11a^−^, CD19^−^, and HLA-DR^−^), and differentiate into the mesenchymal lineages; are essential for proper bone healing and maintenance. Mesenchymal stem cells can integrate the information in their surroundings though mechanotransduction and topography sensing mechanisms, which affect the ability of the MSCs to develop into osteoblasts and eventually osteocytes^1^.

Surface topography can affect cellular functions by influencing the production of proteins that are secreted into the extracellular space to act as signals in the environment. For instance, Schwartz *et al*. demonstrated that higher surface roughness on titanium increased the production of Prostoglandin E2 (PGE2) and Transforming Growth Factor -β (TGF-β)^2^. Furthermore, a comprehensive literature review demonstrated that surface roughness of approximately 4 μm was optimal for osteosarcoma MG63 cells^3^. Protein production is affected by mechanotransduction and these pathways are being modeled to help predict the response of cells to a particular substrate design^4^. Nanofibers have been investigated widely as bone scaffolds. Despite their popularity as bone tissue engineering constructs not many studies have investigated mechanosensing mechanisms involved in transmitting geometry information provided by nanofibers into osteogenic differentiation. Ozdemir *et al*. showed the curvature provided by Poly(methylmethacrylate) (PMMA) fibers recognized by osteoprogenitor cells through RhoGTPases lead to an increase in cellular stiffness resulting in downstream alkaline phosphatase production^5^. The osteoinductive effects have been connected to the dimension and curvature of the nanofibers, as well as the factors that the cells produce without the application of exogenous factors^6–8^.

Osteogenesis involves a complex interplay between various genes that collectively encodes either growth factors or transcription factors to facilitate skeletal development. Much progress have been made throughout the last two decades to explain the genetic and molecular mechanisms for osteoblast and osteoclast physiology^9^. Despite the current knowledge of the molecular players and events that regulate osteogenesis little is known about the initial events that trigger skeletal development and regeneration. Regenerative Engineering holds promise to meet the current need for overcoming the tissue implant barrier, non-union bone repair and dental implant success as well as reducing costs of osteogenic therapies and improving patient comfort.

Biomaterials play significant roles in regenerative therapies and various studies reported significantly increased bone regeneration with fibrous scaffolds^10,11^. The biomimetic nature of fibrous scaffolds has been shown to increase the production of various bone specific extracellular matrix proteins as well as inorganic molecules such as hydroxyapatite (HAp), tricalcium phosphate (Ca_3_(PO_4_)_2_) and composite crystals of both HAp and Ca_3_(PO_4_)_2_. However, the use of biomaterials for regenerative therapies is a relatively new field and detailed investigation is required to elucidate the molecular interactions taking place at the cell biomaterial interface. Several studies reported to explain the global overview of important players in bone regeneration on biomaterial scaffolds, however no groups have yet developed a global model of signaling molecules that drives osteogenicity on synthetic bone regenerative engineering scaffolds.

Loss of function (LOF) experiments are an indispensable part of a research study to gain understanding about the necessity of a target protein or target gene in a scientific problem. The discovery of RNAi^12^ increased the feasibility of LOF experiments. One of the most promising uses of RNAi technology that has transformed biological research is incorporating the genome scale high throughput screens into RNAi methodology to unfold the key genes and proteins of a known or expected phenotype. High throughput screening (HTS) experiments have lead the new area of functional genomics and allowed researchers to study the extent of the genes that are only specific for a certain phenotype of interest. In an RNAi screening experiment a series of assay optimization steps are required to ensure the depletion of protein of interest and overall cell viability. The main steps can be summarized as (1) distributing the library information into HTS plates (either 96 well or 384 well plates), (2) Seeding of cells into the plates with a predetermined density (usually in between 10,000–40,000 cells/ well for a 96 well plate) and introducing the dsRNA (double stranded RNA) construct with the predetermined concentrations of transfection agents and other required conditions for successful entry of the construct into the cell, and (3) the automated detection step to gain quantitative information about the effect of dsRNA^13^. Even though the experimental steps seem straightforward, the assay optimization is relatively cumbersome when compared to other high throughput experiments.

This study assayed the effect of substrate topography, in particular curvature, that is provided by synthetic polymer fibrous scaffolds using high throughput siRNA screening. Further, we assessed the relationship between cellular morphology and the osteogenesis detected on fibrous topography. Several earlier studies reported that signaling cascades affect osteogenesis; however, to date no studies were performed at a high-throughput scale that would show the signaling cascades responsible for osteogenesis on fibrous topography. We used 2 siRNA libraries (a Phosphatase Library that consists of 237 genes and a Kinase library that consists of 636 for a total of 873 genes) to study osteogenic phenotypes observed on smooth PMMA and PMMA fiber scaffolds. A special 96 well plate was developed and optimized for assay conditions. After statistical analysis of the primary screen, a secondary screen was performed to determine the impact of the statistically significant osteogenic targets on cell shape with experimental topographies. The results shed light into molecular mechanisms that specifically play roles in substrate curvature mediated osteogenesis on synthetic regenerative engineering scaffolds.

## Materials and Methods

### Materials

Polymethylmethacrylate (PMMA M_v_ = 120,000, Cat No: 182230), Poly (2-hydroxyethly methacrylate (PHEMA M_v_ = 300,000, Cat No: 192066), tetrahydrofuran (THF, Cat No: 401757), dimethylformamide (DMF, Cat No: D4551), glycerol-2-phosphate sodium salt (Cat No: G9422), ascorbic acid (Cat No: 1043003), Nile Blue Dye (Cat No: N5632) was purchased from Sigma-Aldrich (St Louis, MO). Human SaOS2 cells (Cat No: HTB85) and McKoy’s 5 A Media (Cat No: 30–2007) was purchased from ATCC (Manassas, VA), Heat treated Fetal Bovine Serum was purchased from Atlanta Biologicals (Lawrence, GA) (Cat No: S11050). Monoclonal mouse anti-human RUNX2 antibody (Cat No: sc-390351) was from Santa Cruz Biotechnology (Dallas, TX). In cell western (ICW) reagents; Odyssey Blocking buffer (Cat No: 92750000), LICOR IRDye 800 CW goat anti-mouse secondary antibody (Cat No: 925–32210), LICOR IRDye 680 RD goat anti-mouse secondary antibody (Cat No: 925–68071) were purchased from LICOR (Lincoln, NE).

### Development of Customized HTS Assay Plate with Altered Substrate Topography

In order to unravel the effect of substrate topography using HTS experiments, an in-house HTS culture plate with desired topography was built. The electrospinning method was utilized to create the ECM-like fibrous topography with diameters matching our earlier study^5^. First, 96 well plate black colored plastic grid walls with transparent plastic lids and 0.005” thick clear polystyrene sheets for imaging purposes (Evergreen Corp, Northbrook, IL) were purchased commercially. Polystyrene sheets were cut as 96 well plate bottoms (Fig. S1,Ai). Next, for the flat topography, the polystyrene sheets were spin coated with 2% PMMA dissolved in nitromethane at 5000 rpm for 10 secs. In order to create fibrous topography that could allow cells to spread only on fibers a two-step process followed. First, the polystyrene sheets were spin coated with 2% pHEMA dissolved in 70% EtOH at 3000 rpm for 15 secs to reduce cellular attachment onto surfaces except the fibers. Second, pHEMA coated polystyrene sheets were moved to the electrospinning unit and the electrospinning protocol was followed as described previously in order to keep a consistent diameter^5^. Briefly, 25% w/vol PMMA solution (dissolved in 1:3 THF:DMF solvent mixture) was loaded into a 10 mL syringe, purged using a syringe pump until a polymer solution drop was formed and a 12 kV voltage was applied to form a Taylor cone. The syringe tip to collector distance was set to 18 cm and PMMA fibers were collected onto the pHEMA coated polystyrene sheets for 2 minutes until a very thin monolayer of fibers covered the whole surface. The plate bottoms and tops were sent to Nanofibers Solutions Inc for ultrasonic welding in order to bond the polystyrene sheets with desired surface topography onto the plastic grid walls. In this way, the 96 well plate would have either a flat PMMA coating or PMMA fiber coating at the bottom (Fig. S1,Aii) (Columbus, OH). After HTS culture plates were fabricated, potential leakage between adjacent wells was tested by adding Phosphate Buffer Saline (PBS) or Nile Blue dye (1 mg/mL in PBS) into alternating wells of the HTS plate respectively. The optical density (OD) at 350 nm values of each well for 4 consecutive days were monitored and results were pooled into two groups as PBS and Nile Blue dye containing wells to track possible contamination of one into another (Fig S1,B).

### Determination of Transient RUNX2 Expression Levels

After successful generation of an HTS plate for the study, we next determined transient baseline levels for the osteogenic marker RUNX2 that were to be used in the screening experiment. 100,000 SaOS2 cells were seeded into each well of a 6 well plate; then, following an initial attachment period (16 hours) cell culture media was replaced from growth medium into growth medium supplemented with ascorbic acid and glycerol-2-phosphate sodium salt. At each time point (Day 2, 4, 5, 6, 7, 8) cells were lysed by first discarding the culture medium and rinsing with PBS, adding RIPA buffer (150 mM NaCl, 1% IGEPAL, 0.5% Na Deoxycholate, 0.1% SDS, 50 mM TRIS) and further applying three freeze-thaw cycles. After determining the total protein concentration using UV-vis at 280 nm, cell lysates were denatured and boiled for 10 mins in 6x laemmli buffer and 20 µg total protein was loaded into each well of a 10% SDS-PAGE gel and proteins were separated at 120 V for 3 hours. Separated proteins were transferred onto a PVDF membrane at 100 V for 1 hour in a wet transfer tank with a circulating cooling system. After transfer was complete the PVDF membrane was blocked using Odyssey blocking buffer for 1 hour. The blocked membrane was then transferred into a clean container and diluted primary antibody cocktail (monoclonal mouse RUNX2 (Cat No: sc-390351) 1:100, monoclonal rabbit beta tubulin (Cat No: sc-5274) 1:100) solution was added onto the membrane and incubated overnight in the fridge with moderate rocking. The next day primary antibody was discarded and the membrane was washed with Tris Buffer Saline with Tween-20 (TBST) 3 times for 10 minutes each. Diluted secondary antibody cocktail (IRDye 680RD goat anti-rabbit polyclonal secondary antibody and IRDye 800CW goat anti-mouse polyclonal secondary antibody at 1:200 dilution for each) was incubated with the membrane for 1 hour and western blot was finished by washing three times with TBST and one time with TBS for 10 mins. The membranes were immediately imaged using Odyssey LICOR IR Imaging scanner and the bands were analyzed using LICOR analysis software. RUNX2 expression levels were normalized to tubulin levels. (Fig. S2)

### In Cell Western Blot

LICOR Odyssey Imaging system allows for quantitative determination of intracellular protein expression levels of cells growing in 96 well culture plates. These In Cell Western blotting (ICW) experiments were performed in cells growing in HTS plates transfected with siRNA library constituents. For ICW experiments after the desired culture period, cells growing in HTS plates were removed from the incubator and cells were washed with warm PBS and fixed with 4% paraformaldehyde solution for 15 minutes followed by washing and permeabilizing with 0.1% TritonX100 in PBS 5 times each. Then the plate was blocked using Odyssey blocking buffer for 1 hour and incubated with Runx2 primary mouse antibody (Santa Cruz Biotechnology, Dallas, TX) solution (1:100) for 3 hours at RT with moderate rocking. After washing the wells, cells were incubated with goat anti-mouse secondary antibody (IRDye 800CW) (**1:200)** in 0.1% Tween 20 for 1 hour at RT in the dark. After the incubation was complete the wells were washed 3 times using PBST and once with PBS and further imaged using LICOR Odyssey infrared imaging system.

### Pilot siRNA Transfection Studies

After confirmation of transient RUNX2 expression levels in SaOS2 cells, we next performed a series of experiments to determine the optimum cell seeding density, siRNA transfection methodology and transfection efficiency. In order to prevent oversaturation of fluorescent signal due to high seeding densities 3 different seeding densities were tested. 10,000 cells/well, 20,000 cells/well and 30,000 cells/well were seeded into the wells and allowed to attach for 16 hours. At the day of transfection (experimental day −1) the culture media was changed with the growth media not containing PenStrep due to toxicity initiated by synergy between transfection reagents and antibiotics based on information provided in manufacturers protocols. In order to determine siRNA transfection efficiency, control (only the transfection reagent no siRNA), scrambled (siRNA for sequence of no biological information) and All Star siRNA sequence (an siRNA sequence known to result in cell death) treatments were performed. For transfection, 3 µl of Lipofectamine RNAiMax 2000 (Cat No: 13778075) (Life Technologies, Carlsbad, CA) was aliquoted into 50 µl of OPTIMEM media without Phenol Red (Cat No: 11058021) (Life Technologies, Carlsbad, CA) and mixed with a siRNA solution (3 µl siRNA and 50 µl OPTIMEM) and allowed to incubate 10 min at room temperature prior to addition into cell culture plates. The transfection cocktail was pipetted into designated wells and further moved back into incubator allowing transfection to proceed for 24 hours. After the siRNA transfection is complete (experimental Day 0) the growth media was exchanged back to the media containing PenStrep to eliminate possible contamination. Cells were then incubated for 5 days and Alamar Blue cell metabolic activity assay was performed. Alamar Blue is membrane permeable, non-toxic dye that fluoresces when reduced, which is an indicator of cell viability, metabolism and health. Briefly, 10,000/20,000/30,000 cells/well was seeded into the wells of HTS plates and incubated 5 days (experimental time point for primary screen) before Alamar Blue Reagent (Cat No: R7017) (Thermo Fisher, Carlsbad, CA) was added to the wells. After 1 hour incubation with Alamar Blue reagent, the fluorescence intensity values were measured. 15,000 cells/well seeding density was chosen depending on the color observed in wells after the incubation period. In order to determine the successful transfection, a control siRNA that targets a cell death gene was used. As seen in Fig. S3 cell death gene transfections leads to lowest metabolic activity. A scrambled siRNA sequence was used to understand the effect of transfection process on metabolic activity of cells. As seen in Fig. S3 the metabolic activity of cells transfected with scrambled sequence did not lead to a decrease in metabolic activity.

**Figure 1:**
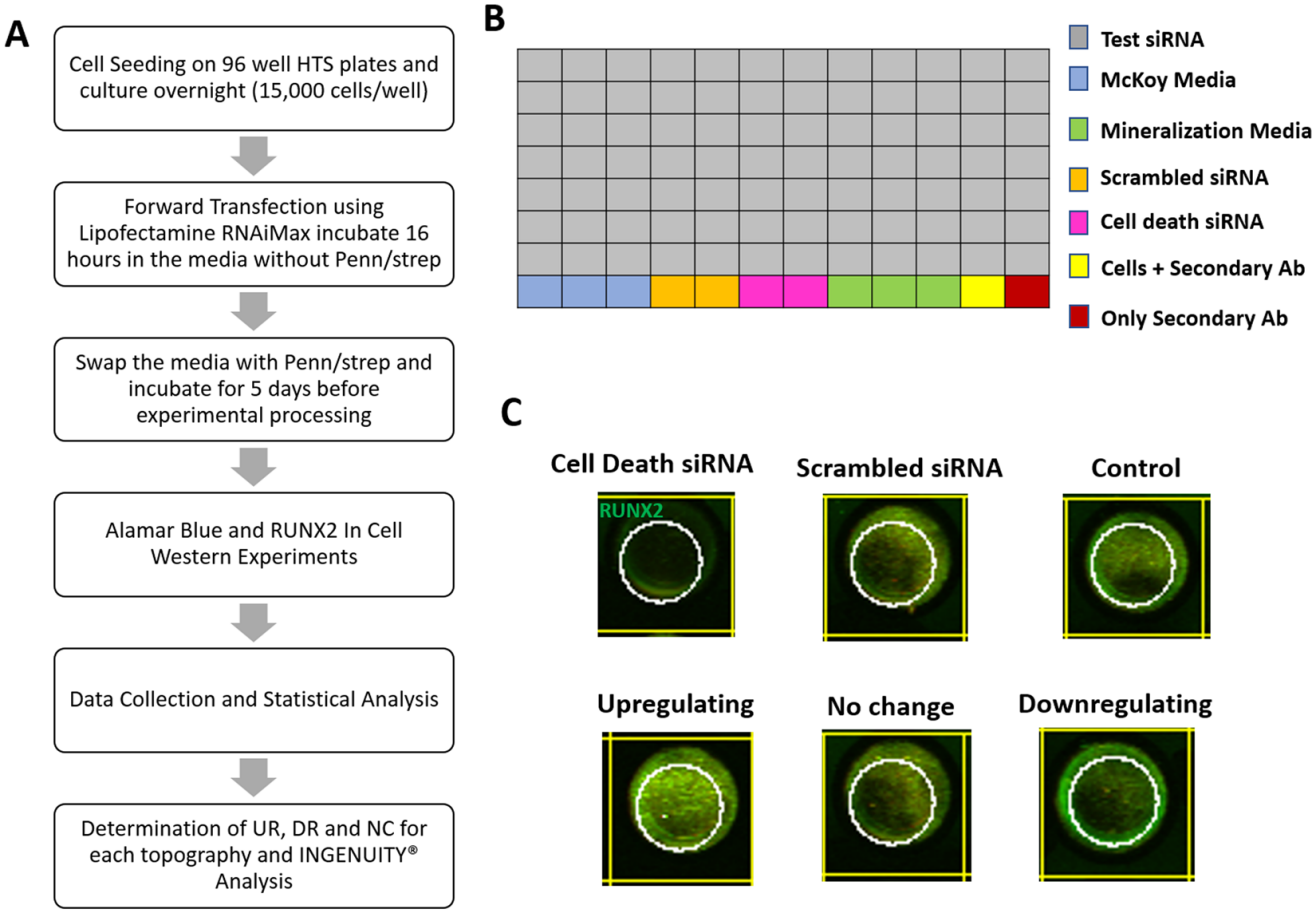
(**A**) Schematic workflow of the primary screen. (**B**) High-throughput siRNA library screen of 863 genes were distributed into designated wells in triplicates in 96 well plates. For each plate, SaOS2 cells grown in McKoy’s media (blue wells), SaOS2 cells grown in mineralization media (green wells), scrambled siRNA (orange wells), cell death siRNA (magenta wells), SaOS2 cells with only secondary Ab (yellow well) and only secondary Ab (red well) wells were used as internal controls. Three 96 well plate replicate was used. (**C**) Representative ICW images for Cell death, Scrambled, Mineralization Media Control, A test gene that upregulated osteogenesis, A test gene that downregulated osteogenesis, A test gene that has no effect osteogenesis compared to control. White circles represent the region of interest for quantification of RUNX2 ICW signal.

**Figure 2:**
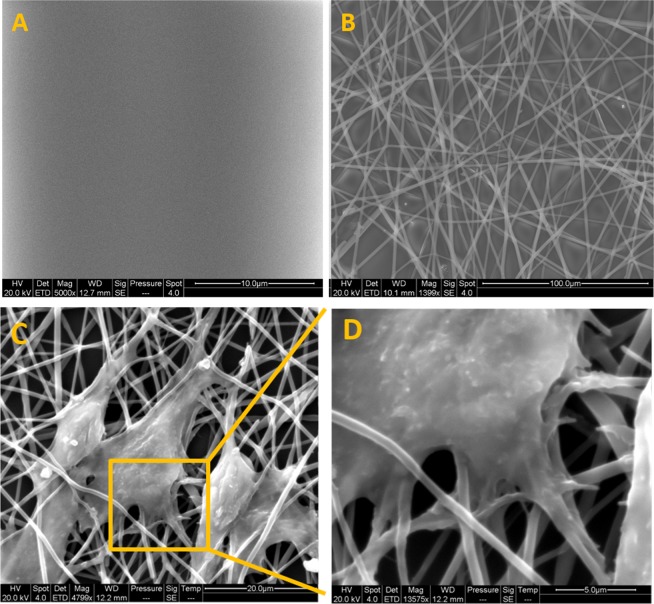
SEM images of smooth (**A**) and fibrous (**B**) topographies covered polystyrene sheet show fibers are sparsely distributed to allow cell spreading yet still create a fibrous topography over the flat underlying polystyrene layer. Cellular spreading and cell fiber interactions shown in C and D. Cells possess a spreading morphology (**C**) and present adhesive extensions that are wrapping around single fibers (**D**).

**Figure 3:**
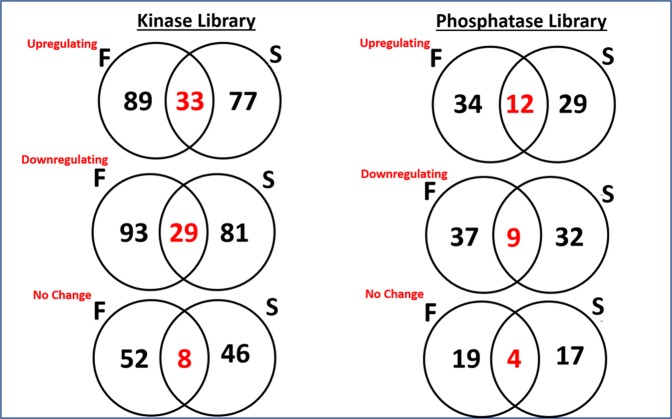
Summary of the filtered genes are shown in the diagram for each library (kinase and phosphatase). The unbiased selection criteria used in the statistical analysis step allowed distinguishing of upregulating, downregulating and no change targets for each topography. There were a significant number of intersecting genes in both smooth and fiber topography categories. A two sample t-test statistical analysis was performed to distinguish the different genes in this category.

### High Throughput siRNA Library Screen Overview

An siRNA library of 863 human genes consisting of selected intracellular signaling kinases and phosphatases was used to identify osteogenic phenotypes associated with corresponding genes in cells growing on flat or fibrous topographies. Kinases are proteins with an ability to add phosphate groups to the signaling proteins which then switch on certain function within intracellular signaling and phosphatases are known to remove phosphate groups that switch off the function accordingly^14^. We therefore could gain information about the on/off state of certain signaling pathway by screening all the kinases and phosphatases. A detailed list of genes used in this study is listed in Supplementary File 1. The siRNA Library used in this study was aliquoted from the source library at Penn State Hershey Medical School High Throughput Screening Facility (Hershey Medical School, Hershey, PA). The library for Human Kinase and Phosphatase was obtained from Life Technologies® Stealth Library (Thermo Fischer Scientific, Carlsbad, CA). For targeted siRNA mediated silencing, 3 individual siRNA sequences per gene were added into the wells containing cells growing on different topographies. Five days after gene silencing the cells were assayed for osteogenic differentiation. The osteogenic phenotype of SaOS-2 cells were assessed by quantifying the protein expression levels of a well identified transcription factor RUNX2^15^. Alamar Blue dye reduction analysis was performed to determine the cell viability. After filtering out the genes that affect cell viability, remaining targets were then organized into upregulating (the targets that upregulate osteogenicity), down regulating (the targets that downregulate osteogenicity) and no change (the targets that do not influence osteogenicity) groups for flat and fibrous topographies. The upregulating targets on fibrous and flat surfaces were further enriched using INGENUITY software and a comparison heatmap of the signaling pathways that are significantly upregulated and downregulated on different topographies were represented. To detect the genes that significantly upregulate, downregulate or do not change (Nochange) osteogenesis on both fibrous and flat surfaces, a two sample t-test was performed. After obtaining the list of significant genes, the top 10% of each category was selected for a secondary screen. The second siRNA screen was run against the significant targets on fibrous and flat surfaces and the adhesion phenotype of the targets were assessed by immunofluorescence microscopy and cell morphology analysis.

### Cell Culture and siRNA Mediated Transfection

SaOS-2 cells (ATCC, Manassas, VA) were cultured using McKoy’s Essential Medium (ATCC, Manassas, VA), 15% fetal bovine serum (FBS) (Atlanta Biologicals, Lawrenceville, GA), 1% penicillin-streptomycin (Pen-Strep) (Life Technologies, Carlsbad, CA) at 37 °C, 5% CO_2_ with 95% humidity. When the cells reached 80% confluency, cells were trypsinized using 0.25% Trypsin 0.15 mM EDTA (Invitrogen, Carlsbad, CA) and either replated for culture expansion or further treatment to prepare cell suspensions for experiments. For siRNA screening experiments, growth medium containing additional 10 µg/ml ascorbic acid (Sigma Aldrich, St Louis, MO) and 3 mM glycerol 2-phosphate sodium salt hydrate (Sigma Aldrich, St Louis, MO) was used and cells were grown for 5 days at 37 °C. To sterilize HTS plates prior to siRNA transfection, plates were UV sterilized then washed twice with growth media (containing PenStrep) in order to remove possible residual solvents and impurities due to materials processing. 15,000 cells/well were seeded, let sit 30 minutes at room temperature inside the laminar flow cabinet to reduce edge effects^16^, then moved into a cell culture incubator (37 °C, 5% CO_2_ with 95% humidity) and allowed to spread overnight. At the day of transfection (experimental day -1) the culture media was changed with the growth media not containing PenStrep due to toxicity initiated by synergy between Lipofectamine2000 and PenStrep based on manufacturers protocols. 3 µl of Lipofectamine RNAiMax 2000 (Life Technologies, Carlsbad, CA) was aliquoted into 50 µl of OPTIMEM (Life Technologies, Carlsbad, CA) and mixed with a siRNA solution (3 µl siRNA and 50 µl OPTIMEM) and allowed to incubate 10 min prior to addition into cell culture plates. The transfection mixture was pipetted into designated wells in HTS plates (Fig. S1) then moved back into the incubator and the transfection proceeded for 24 hours. Transfections were performed in triplicates for statistical testing of the quantitative assay results. After the siRNA transfection was complete (experimental Day 0) the growth media was exchanged back to the media containing PenStrep to eliminate possible contamination. The completion of transfection was monitored in control wells that contained an siRNA sequence for a gene that leads to cell death. Cells were then incubated for 5 days and media was refreshed every 2 days. The possible effect of transfection on cell metabolism was measured by reduction of Alamar Blue Dye (Life Technologies, Carlsbad, CA). At the morning of day 5, an Alamar Blue dye solution was mixed with cell culture media to a final concentration of 10% and added into empty wells containing cells and incubated for 2 hours at 37 °C and then fluorescence intensity of the plates were read on a plate reader set to measure absorbance at 530/590 nm.

### Analysis of Osteogenic Phenotype on Substrate Topography

The osteogenicity was monitored by quantifying relative protein expression levels of Runx2 with an ICW experiment. Briefly, after reading the Alamar blue levels, the media with alamar blue was discarded from each well, then the cells were washed with warm PBS and fixed with 4% paraformaldehyde solution for 15 minutes followed by washing and permeabilizing with 0.1% Triton X-100 in PBS 5 times each. Then the plate was blocked using Odyssey blocking buffer for 1 hour and incubated with Runx2 primary mouse antibody (Santa Cruz Biotechnology, Dallas, TX) solution (1:100) for 3 hours at RT with moderate rocking. Well H12 in the 96 well plate (Fig. 1B) was assigned as a control well and incubated with blocking buffer without primary antibody and further used to correct background. After washing the wells, cells were incubated with goat anti-mouse secondary antibody (IR 800CW) (**1:200)** and 0.1% Tween 20 for 1 hr at RT in dark. After the incubation was complete the wells were washed 3 times using PBST and once with PBS and further imaged using LICOR Odyssey infrared imaging system.

### Data Analysis and Normalization

Data analysis is automated in an R script to parse and normalize the Alamar Blue and RUNX2 raw data. We started our data analysis with Alamar Blue results and first eliminated the background from all data. Then we performed an initial normalization for edge effects to eliminate noise coming from the outermost wells in each plate that is independent from siRNA reagent^17^. In order to correct for positional effects, a correction factor was calculated for each plate by dividing the trimmed mean (20%) of internal wells by the trimmed mean of edge wells. Values from wells located at plate edges were multiplied by this correction factor, as plate normalization, then fold change values were calculated. The plate to plate variability was normalized to the trimmed mean (20%) of all area values from the plate excluding positive controls. Fold change relative to mock-transfected control were calculated by dividing the plate-normalized values by the median of all the normalized mock transfected controls from all plates. After Alamar Blue normalization, outliers were filtered as follows. We first plotted the distribution curve of Alamar Blue values, and then deleted all negative values in the left tail and top 10% of highest values in the right tail (according to our calculations about 10% of the values were negative, that’s why we picked the top 10% of positive tail). After outlier filtering 519 genes in fiber, 490 genes in flat for Kinase and 199 genes in fiber, 186 genes in flat for phosphatase library remained to be analyzed. Next, we filtered for the unhealthy cells. For this, we plotted both the sample and the control distributions and we observed that the minimum value of controls are around 0.5 so we deleted the genes that have Alamar Blue scores less than 0.5. After Alamar Blue score filtering, 304 genes in fiber, 275 genes in Smooth for Kinase and 115 genes in fiber, 103 genes in Smooth for phosphatase library remained to be analyzed. The wells for these filtered gene sets were then analyzed for osteogenesis. To correct the RUNX2 readings for the cell number, we normalized RUNX2 values to corresponding Alamar Blue values and partition the genes into three categories. We defined the top 40% of the positive tail of the data as an upregulated region (shown as UR), the bottom 40% of the lower tail as the downregulated region (DR) and anything in between as the nochange region (NC) as compared to scrambled siRNA transfection control. The UR and DR genes for each topography were separated from the data pool for further enrichment and INGENUITY analysis. We were interested in the intersection of two regions in fiber and smooth representing the genes that were responsible for osteogenesis on both fibrous and flat substrates. (There are three intersection sets UR, DR and NC). In each intersection group, a two-sample t-test was performed to identify genes which were differentially expressed between two different substrates. Due to small sample size, we used permutation to calculate the t-test p value. For each permutation, we randomly shuffle the fiber/smooth label, and then used the permuted samples to calculate the test statistic. We repeated this procedure B = 1000 times and get 1000 permuted t-test statistics. Then we compared those 1000 permutation based statistics with our original t-test statistic. The t-test p-value was calculated as follows;

$${p}_{j}^{\ast }=\frac{1}{B}\#(|{t}_{j}\,(b)|\ge |{t}_{j}|)$$

Where ${p}_{j}^{\ast }$ is the p-value for the jth gene, $|{t}_{j}\,(b)|$ is the bth permuted t-statistic and $|{t}_{j}|\,$ is the original t-statistic. That is, the p-value is the proportion of the permutation based statistics that have a larger absolute value than the original statistic. We obtain multiple p-values in each intersection group. In order to control the family-wise error rate, we applied the Bonferroni procedure for the multiple testing correction^18^.

### INGENUITY Pathway Analysis

Protein interaction networks for UR, DR gene sets were generated using INGENUITY Pathway Analysis (IPA) (Ingenuity Systems, Redwood City, California). Initially the UR, DR and NC target lists (Supplementary File 2) were uploaded into IPA and signaling network maps involving the HTS hits were obtained. The Network maps for Smooth vs Fiber topographies were compared side by side as shown in Fig. S4. This method allowed us to compare the number and location of hits within the corresponding signaling cascade. Since we ran the whole Kinase and phosphatase genes in HTS for the study, we further decided to compare all the hits within all the signaling cascades using IPA. We utilized the comparison feature of IPA which compares two or more omics data files based on the targets’ expression levels. After our analysis we had a list of targets in three categories (UR, DR and NC) and we wanted to compare these targets for smooth and fiber topography. We generated a new folder for IPA to pull out our targets inside the signaling cascades and compare between topographies. Therefore, we merged all the library hits into one import file by assigning pseudo fold-change and p-values. We defined p-values 0.00001 for anything that was observed as significant in two sample t-test and 1 for anything that wasn’t. Thus, any upregulated target was assigned to have a fold change of 2 and any downregulated target assigned to have a fold-change of −2. Afterwards all the assigned targets were merged into an excel file and the file was used as a source file for IPA Comparison Analysis. The Comparison Heat Map was produced separately using the R statistical package.

### Fluorescence Microscopy

15,000 cells/well were plated to the different substrates with and without selected siRNAs. 48 hours after transfection, the cells were fixed and stained for focal adhesions, stress fibers and the nucleus following our standard immunostaining protocol. Briefly, samples are fixed in 4% paraformaldehyde for 15 min following a wash in cold cytoskeletal stabilizing buffer to remove unbound proteins for 1 minute. Blocking with 5% BSA in PBS for 45 mins is followed by application of the primary antibody for 1 hour. 3 washes in PBS are followed by the secondary antibody for 1 hour and another set of 3 washes. If DAPI or Phalloidin were applied they are incubated on the samples for 30 min at 1: 10,000 and 1: 5,000. Images were acquired on a Leica DM5500 B microscope with a Leica DFC360 FX camera run by LAS 3.7 software (Leica, Buffalo Grove, IL). The phalloidin images were quantified for shape and intensity parameters. The CellProfiler^19^ software automates this process and allows for monitoring of the results to feed back into the process. Several factors were compared within the results provided by the shape analyses of the cell boundaries as defined by the cytoskeleton. The compactness is defined as the variance of the radius divided by the area. The extent is the area divided by the area of the bounding box, while the $form\,factor=\frac{4\pi \times area}{perimete{r}^{2}}$. More detailed explanations of the parameters calculated can be found in the online CellProfiler documentation (http://cellprofiler.org/). Mean values were compared by Analysis of Variance with a Fisher’s test. Significance was taken when p < 0.05 for the comparison against the flat surface control for that individual siRNA.

## Results

### Overview of the High-throughput siRNA Library Screen on Altered Topography

The overview of the primary screening steps is displayed in Fig. 1A. The experiment was conducted in triplicate and as shown in Fig. 1B each plate had proper controls for the data analysis described in the Materials and Methods section. Triplicate plates ensured the proper validation of off-target effects such as background signal from the secondary antibodies or non-specific binding of secondary antibodies to the cell membrane. In addition, scrambled siRNA and Universal cell death gene wells ensured the proper administration of siRNA into the cells. The ICW experiment targeting RUNX2 as the osteogenic target was quantified using LICOR Odyssey scanner as shown in Fig. 1C. RUNX2 signal shown as green is altered in upregulating genes and diminished in downregulating genes compared to control.

### Cell Morphology and Spreading on Fibrous Scaffolds

Osteogenic phenotype changes in response to altered topography were monitored by generating synthetic fibrous matrices using electrospinning. PMMA was used as a generic polymeric material because it is widely accepted in tissue engineering applications due to its low immunogenic and cytotoxic response and low cost. To exclude the material related biases and ensure that the observed response was only due to surface topography we used flat, spin coated PMMA as a material control. Fig. 2A, B shows each type of topography used in this study. The diameter of the fibers was measured as 0.938± 0.304 µm. In addition, Fig. 2B, C shows the SEM images of cell morphology and spreading on fibrous surfaces. Cells not only elongate the cell body along the fiber layer underneath but also generate microextensions wrapped around the fibers.

### Osteogenic Genes Based on Primary Screen Analysis

A total of 863 genes were screened during the study consisting of two separate libraries representing human kinases and phosphatases. After filtering and data analysis, genes that followed our selection criteria are shown in Venn diagram (Fig. 3). In the Kinase library 122 genes were upregulated on fibrous substrates and 33 out of 122 were both upregulated on fibrous and smooth substrates, and 77 genes are only upregulated on smooth surfaces indicating a positive relationship between their upregulation and osteogenesis. Our selection criteria also filtered 203 downregulating genes 29 of which downregulated on both topographies and 110 genes on smooth surfaces. We found 60 genes that have no effect on osteogenesis on fibrous topographies accompanied with 55 genes on flat smooth surfaces.

In the Phosphatase library, 46 genes were upregulated on fibrous surfaces, 12 out of 46 were both upregulated on fibrous and smooth surfaces, and 41 genes were upregulated on smooth surfaces indicating a positive relationship between their upregulation and osteogenesis. Our selection criteria also filtered 46 downregulating genes, 9 of which downregulated on both topographies and 41 genes on smooth surfaces. We found 23 genes have no effect on osteogenesis on fibrous topographies accompanied with 21 genes on flat smooth surfaces.

### INGENUITY Pathway Analysis of Substrate Topography

**Figure 4:**
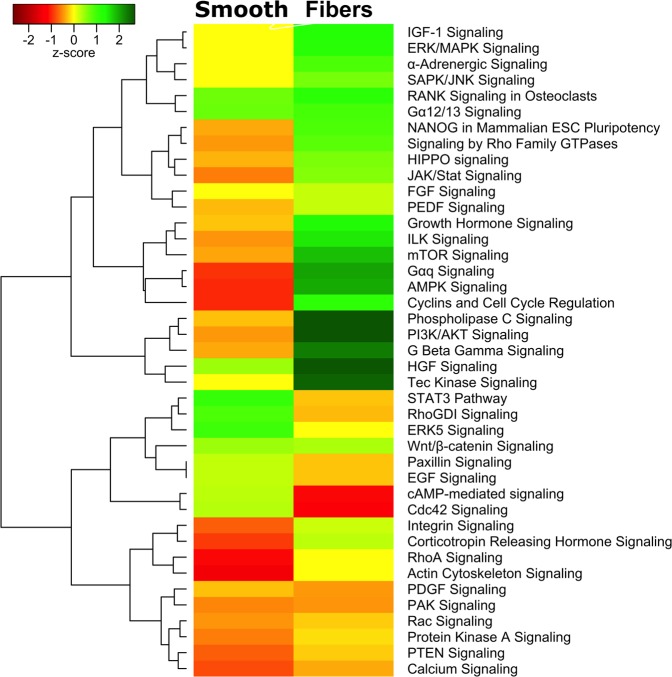
Comparison Heatmap comprised of the total number of HTS hits for each topography within the specified signaling cascade using INGENUITY Comparison function. Green color indicates the number of upregulated signaling molecules were higher within the particular signaling cascade and red indicates number of downregulated signaling molecules were higher within the particular signaling cascade.

The genes that were sorted in Fig. 3 were then enriched to identify the significant signaling pathways on fibrous and smooth topographies. INGENUITY results were organized in a heatmap to show the upregulated and downregulated signaling pathways on fibrous vs flat substrates respectively (Fig. 4). The signaling pathways that were significantly upregulated on fibrous substrates were listed as mTOR Signaling, AMPK Signaling, Cyclins and cell cycle regulator and ILK Pathways. Another set of signaling pathways that were found to be upregulated were NANOG, RhoA GTPase, HIPPO, Jak/STAT, PI3K/AKT, Phospholipase C and Tec Kinase. Whereas the downregulated signaling pathways were listed as cdc42, Rac1, PTEN, STAT3, PAK, Protein Kinase A, Calcium Signaling and PDGF. The heatmap provides a global picture of Signaling Pathways that play a role on substrate topography guided osteogenesis.

### Effect of Significantly Upregulated Genes on Cell Adhesion Phenotype and Morphology

**Figure 5:**
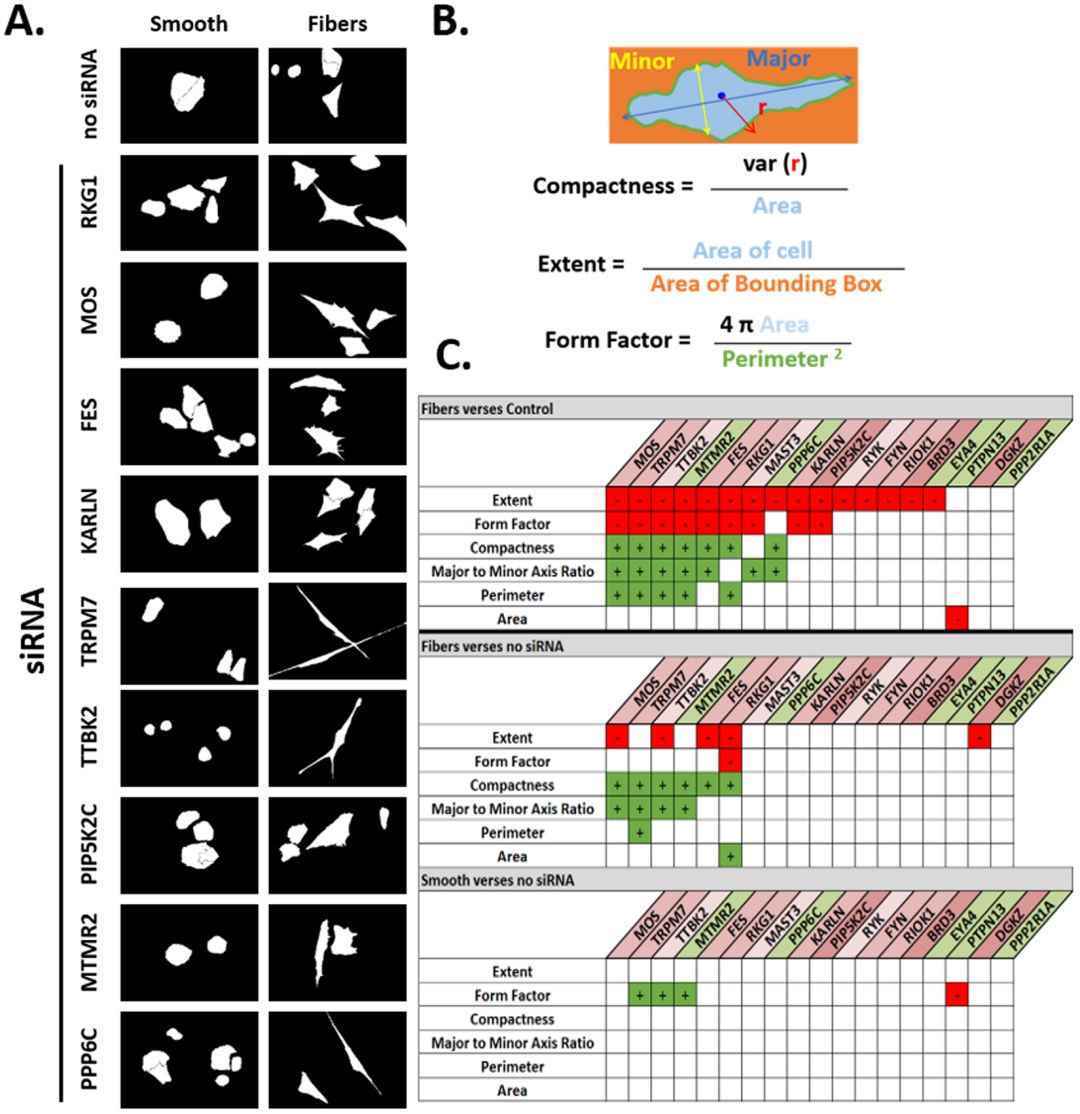
Morphological characteristics of cells treated with siRNA. (**A**) The shape of the actin cytoskeleton is represented by thresholded images after automated segmentation. Representative images shown for the indicated siRNA after thresholding. (**B**) The compactness, extent and form factor are derived from the primary parameters as indicated in the example cell diagram. (**C**) Morphological parameter classification based on the up or down regulation of the parameter only when the comparison was statistically significant. (p < 0.05 by ANOVA with Fisher’s test). Green and red indicate up or down significance respectively within an siRNA treatment (n = 5 to 26, mean n = 11 ± 4.3 cells per group).

Finally, we ran a secondary scale siRNA screen to determine the effect of significantly upregulated genes on cell adhesion and cellular morphology by immunostaining. RK6, TRPM7, FYN, KARLN, PPP6C and EYA4 demonstrated an increase in the size of focal adhesions (by an increase in relative intensity of the vinculin signal). In contrast, FES, RIOK1, MTMR2, TTBK and PTPN13 showed a decrease in focal adhesion formation (Table 1). Immunostaining of focal adhesions and F-actin filaments showed distinct morphological differences in cell adhesion phenotypes on fibrous surfaces (Fig. 5A).

**Table 1:** Detailed information about the targets selected for the secondary screening after two sample t-test analysis.

	Kinase	Phosphatase
DownRegulated	PRKG1: Serine/threonine kinase that acts as a mediator of nitric oxide (NO) signaling. Phosphorylation of RhoA by PRKG1 blocks the action of contraction, vesicle trafficking reduction of myosin light chain kinase phosphorylation^32^.	EYA4: Transcriptional co-factor, it is a functional phosphatase. Loss of EYA4 function results in increased apoptosis and genetic stress^33^.
	MOS: Serine/threonine Kinase located in the cytoplasm and important in chromatin organization during cell cycle^34^.	MTMR2: It is a phosphatase that acts on chemical messengers that help regulate processes such as transport of fats and proteins within the cell^35^.
	MAST3: serine/threonine Kinase which decrease the NF Kappa B activity and indirectly regulates inflammatory function^36^.	
	FES: Tyrosine kinase. It is downstream of Kit and B1 integrin receptors. It has an N-terminal BAR domain which localize underneath the membrane and sense substrate topography^37^.	
	PIP5K1C: Type I phosphatidylinositol 4-phosphate 5-Kinase. The encoded protein catalyzes phosphorylation of PI4P, found to play role in endocytosis and cell migration^38^.	
	BRD3: Bromodomain extra terminal domain protein is an epigenetic reader of acetylated lysine tail residues. Important in skeletal myogenesis^39^.	
NoChange	KALRN: Serine/threonine kinase promotes the exchange of GDP by GTP. Activates specific Rho GTPase family members. Induces lamellopodia^40^.	PPP2R1A: Serine/threonine-protein phosphatase. This serves as a scaffolding protein. Plays roles during cell cycle through affecting centromeric organization^41^.
	RI0K1: Serine threonine kinase and an ethyl transferase adaptor protein that targets the substrates for demethylation. The encoded protein is essential for the last steps in the maturation of 40S subunits^42^.	
Upregulated	DGKZ: Zinc-finger nuclear kinase thought to be downstream of PKC alpha and mTOR pathways. And important in cell differentiation^43^.	PPP6C: Serine/threonine phosphatase. A negative regulator that restricts G1 to S phase progression. Downregulate MAP3K kinase activation of the IL1 signaling pathway^44^.
	RYK: Tyrosine kinase, important for the WNT-5a dependent induction of MMP2 and cell migration^45^.	PTPN13: Tyrosine phosphatase. Found to interact with Fas receptor and associated with apoptosis^46^.
	TTBK2: Seine/threonine kinase that putatively phosphorylates tubulin^47^.	
	TRPM7: Both serine/threonine kinase and ion channel regulating kinase. Its kinase activity is essential for the channel function. May be involved in a fundamental process that adjusts plasma membrane and phosphorylates Annexin A^48^.	
	FYN: Src family tyrosine kinase involved in the regulation of cell adhesion and motility through phosphorylation of B-Catenin and Delta-Catenin. Effects cytoskeletal remodeling by phosphorylating actin regulator WASP and microtubule associated protein (MAP)^49^.

The morphological shape of cells arises from a combination of factors including the shape of the adhesive surroundings and the state of intra-cellular signaling. In order to understand the effect of a select set of siRNAs on the shape of cells we treated the cells with the indicated siRNAs and then imaged the fixed stained specimens (Fig. 5A). The images were quantified using a semi-automated process in the open-source CellProfiler software environment to produce measures of the shape including major axis, minor axis, perimeter, area, compactness, extent, and form factor (Fig. 5B).

The quantifications were visualized by categorizing the statistically significant differences between treated and untreated substrates as a positive or negative change (Fig. 5C). Mean CellProfiler shape parameter values are represented as bar graphs in Fig. S5. Extent and form factor were mostly decreased while compactness, major-to-minor axis ratio and perimeter were mostly increased in comparison. The greatest number of siRNAs produced significant changes in the fibers verses flat comparison (siRNA treated, Fig. 5C upper). The least number of significant differences were when comparing the treated and untreated groups on the smooth control surface (Fig. 5C lower). siRNAs MCS, TRPM7, TTBK2, MTMR2 and FES displayed similar increase and decrease patterns on the fibers when compared to a flat and untreated case (Fig. 5C upper and middle).

## Discussion

In the present study, we used high-throughput RNAi screening to identify human genes involved in osteogenic differentiation on flat and fibrous topographies. In addition to identifying the signaling pathways involved, our study for the first time showed the genes involved in cell shape and cytoskeletal arrangement on fibrous topography. We quantitatively assessed the morphological parameters of the cells with genes knocked down as identified by our initial screening experiment. Several earlier studies showed that cell shape can drive mesenchymal stem cell differentiation^20^. It was shown, by earlier studies that cytoskeletal proteins, focal adhesions, transmembrane channels and BAR domain proteins were main players to impact cell shape and this further translates into intracellular signal activation. However, the bigger picture describing the connection between cell shape and the signaling proteome was missing. High throughput techniques such as microarrays or whole genome sequencing provide valuable information for identifying genes that are involved in certain cellular phenotype but they cannot provide deep level information on how each of those targets affect cellular phenotype. High throughput siRNA screening is a valuable tool to provide information about the cellular phenotype after knocking down the target gene and this kind of information is provided only by loss of function (LOF) studies^17,21,22^.

Biochemically modified synthetic materials provide flexibility and precision for targeted regenerative therapies. The architecture provided with biomaterials also have significant impact on cellular responses^1^. Substrate curvature, specifically out of plane curvature, is experienced by cells that are attached to collagen filaments and bundles. This shape can be mimicked by electrospun nanofibers. By reducing the diameter and therefore the effective curvature presented to cells, Jaiswal and Brown have shown that the activation of ERK and p38 is diameter dependent^7^. In pushing to further understand the signaling pathways that are involved in materials based osteogenesis our group has shown dependence on RhoA/ROCK and Myosin IIa^5^ and the curvature sensing interactions of POR1 and Arf1 with osteogenesis regulating Rac1^8^. The results presented in this paper give a global overview of signaling proteomes that operate during bone differentiation on nanofibrous substrates and further investigates which signaling proteins were responsible for connecting the curvature sensing through cell shape and osteogenesis.

In our study, we showed mTOR, AMPK, Integrin linked kinase (ILK) signaling cascades were significantly active on fibrous topography. In addition, Cyclins and cell cycle regulation signaling pathways were among the highest difference between fibrous and flat topography. In 2017, Lee *et al*. showed the importance of nanotopography on cell cycle regulation of adult mesenchymal stem cells and provided evidence on activation of Mitogen activated protein kinase (MAPK) signaling using a whole RNA sequencing approach and analyzing data with INGENUITY^23^. In our study we showed on fibrous topography, cell cycle signaling was upregulated at Day 5 as well as ERK1/2, JNK/SAPK signaling cascades also being upregulated. The analysis also indicates several developmentally relevant signaling pathways to be active such as, NANOG in Pluripotency, HIPPO and Wnt/β Catenin. Among the likely candidates HIPPO signaling is a developmentally relevant pathway directly downstream of YAP/TAZ signaling which is a mechanosensitive protein couple^24^. We have not found evidence of studies investigating the role of HIPPO signaling on curved topographies. Thus, HIPPO signaling will be one of the future candidates for our investigation. While RhoGTPase signaling and ILK were activated cdc42, Rac and calcium signaling were downregulated. Several earlier studies showed the importance of substrate topography on activation of RhoA GTPase protein^25^.However RhoGTPases, especially RhoA, Rac and cdc42, operate in a counteracting manner. For instance, when cytoskeletal tension increases the RhoA levels elevate, in contrast when cells extend the cytoskeleton in migration prior to RhoA initiated contractility, Rac1 and cdc42 levels increase^26^. Another set of signaling pathways that are active on fibrous topography is membrane associated signaling pathways. Among those, Phospholipase C, PI3K/AKT, and G Beta Gamma were significantly upregulated on fibrous topographies. Our earlier studies and ongoing work also focus on membrane related mechanisms on curvature sensing. In 2015, we showed while the cytoskeletal tension is increased on curved topographies, the membrane becomes more disorganized and unstable. Using time-correlated single photon counting (TCSPC) we showed the discrepancy between cytoskeletal tension and membrane tension^27^. The wealth of information obtained by our study will shed light on undiscovered pathways that are involved in curvature sensing and this will be useful in both cell mechanobiology and tissue engineering.

High-throughput techniques provide large amounts of useful information, but many measurement techniques such as flow cytometry, arrays and ELISAs do not provide morphological information. Semi-automated image analysis programs have allowed for non-biased analysis of morphological characteristics in a large number of images. Holle *et al*. quantified and found no difference in cell area or eccentricity in cells that had been treated with siRNA for mechanosensitive candidates vinculin, p130Cas, Filamin, SORBS1 (Ponsin), SORBS3 (Vinexin) and control protein paxillin^28^. In contrast Unadkat *et al*. studied cells grown on oxygen plasma treated surfaces and found significant differences in the cell perimeter and nuclear form factor and were able to use a forward feature selection model to correctly classify cells into the surface treatment groups based on nuclear texture, intensity of actin cytoskeleton staining, and cell perimeter^29^. Similarly Neto *et al*. found a difference in area across various multilayer films^30^. Hulshof *et al*. compared micro and nano topographic patterns and found that on the nano-topographic patterns the nuclear orientation was most affected while the nuclear shape parameters were also affected on the micro-topographic patterns. Furthermore, on the nano-topograhic patterns morphological parameters related to the cell area such as perimeter and diameter were affected to a greater degree by a change in the pattern^31^. Beyond the depth of knowledge acquired by these studies, our study assembled the first comprehensive list of mechanosensitive signaling pathways for cells that were provided differential extracellular cues that change the morphology of the cells. Collectively these studies suggest that gene expression is linked to morphological changes.

## Supplementary information

Supplementary Information

## Data Availability

The datasets generated and/or analyzed during the current study are available from the corresponding author on reasonable request.
