# Supplementary material for: Identification of Key Signaling Pathways Orchestrating Substrate Topography Directed Osteogenic Differentiation Through High-Throughput siRNA Screening
Source: Sci Rep. 2019 Jan 30;9:1001. doi: 10.1038/s41598-018-37554-y (PMC6353928; doi:10.1038/s41598-018-37554-y)
Supplement: Supplementary file 1 — Supplementary Information [file 41598_2018_37554_MOESM1_ESM.pdf]

Identification of Key Signaling Pathways Orchestrating Substrate Topography Directed Osteogenic Differentiation Through High-Throughput siRNA Screening.

Tugba Ozdemir[1], Daniel T. Bowers[2], Xiang Zhan[3], Debashis Ghosh[4], Justin L. Brown[2] *

[1]Department of Genetics and Bioengineering, Gaziosmanpasa University, Tokat, Turkey
[2]Department of Biomedical Engineering, The Pennsylvania State University, University Park, PA
[3]Department of Public Health Sciences, Penn State College of Medicine, Hershey, PA
[4]Department of Biostatistics and Informatics, Colorado School of Public Health, Aurora, CO

*To whom correspondence should be addressed:
Justin Lee Brown, Ph.D.
Department of Biomedical Engineering, 205 Hallowell Building, The Pennsylvania State University, University Park, PA, USA 16802-4400, Phone: +1-814-865-5190, Email: jlbbio@engr.psu.edu

Keywords: topography, siRNA, high-throughput screening, osteoinduction, electrospinning, nanofiber

Supporting Information

Fig S1. Development of customized HTS Assay plate with altered substrate topography

Brief overview of the preparation of screening plates and quality control testing before the primary screen. A) (i) 96 well plate tops and lids purchased separately and (ii) polystyrene sheets deposited with electrospun fibers (ii) then placed under the plate grid (iii) and further processed with ultrasonic welding at 90 psi (iv). B) Possible leaking from the wells were tested by adding nile blue into alternating wells of the plate and the dye absorbance in each well measured for 4 days.

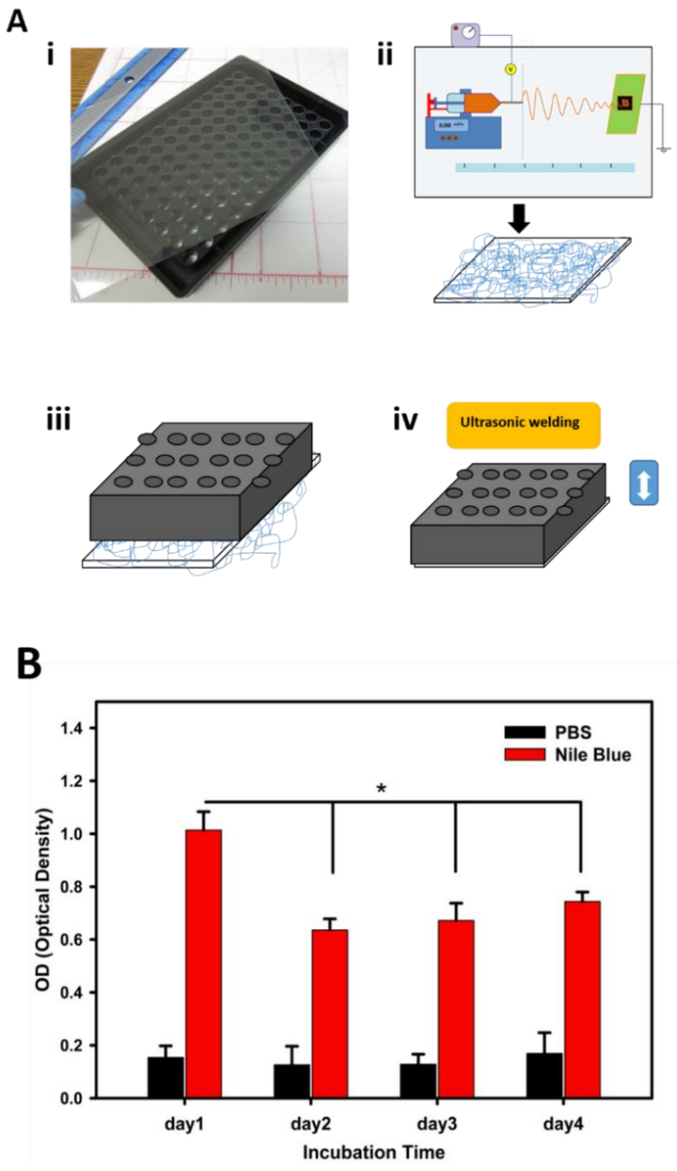

Fig S2. Determination of Transient RUNX2 Expression Levels

The osteogenic marker RUNX2 expression was semi-quantified using western blotting. Runx2 bands appear green and normalized to the house keeping protein tubulin (red). * indicates p<0.05 after a t-test performed comparing to Day 2 sample.

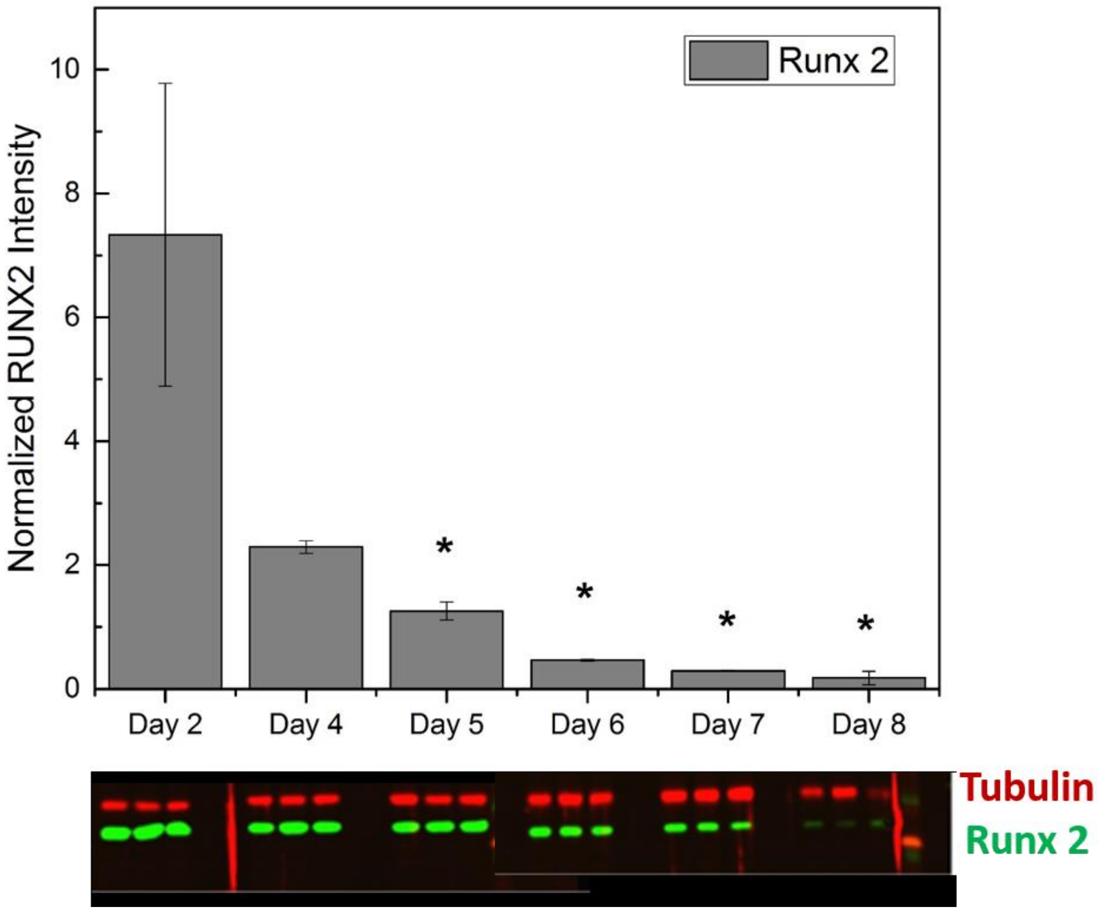

Fig S3. Pilot siRNA transfection studies.

Qualitative determination of cell seeding density based on the alamar blue signal intensities at Day5. Next, siRNA off-target effect was examined using scrambled siRNA sequence and All Star sequence which is a siRNA sequence results in cell death upon successful administration.

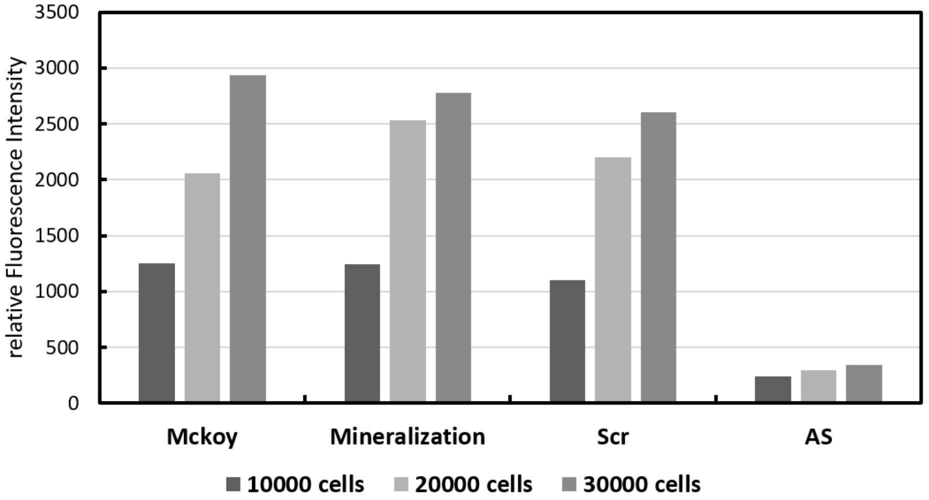

Fig. S4. Side-by-side Comparison Signaling Network Enriched using INGENUITY®

The significant genes after screening data analysis were uploaded in INGENUITY software. Image shows side-by-side representation of genes (framed in purple) in Rho GTPases signaling for fiber and smooth topographies.

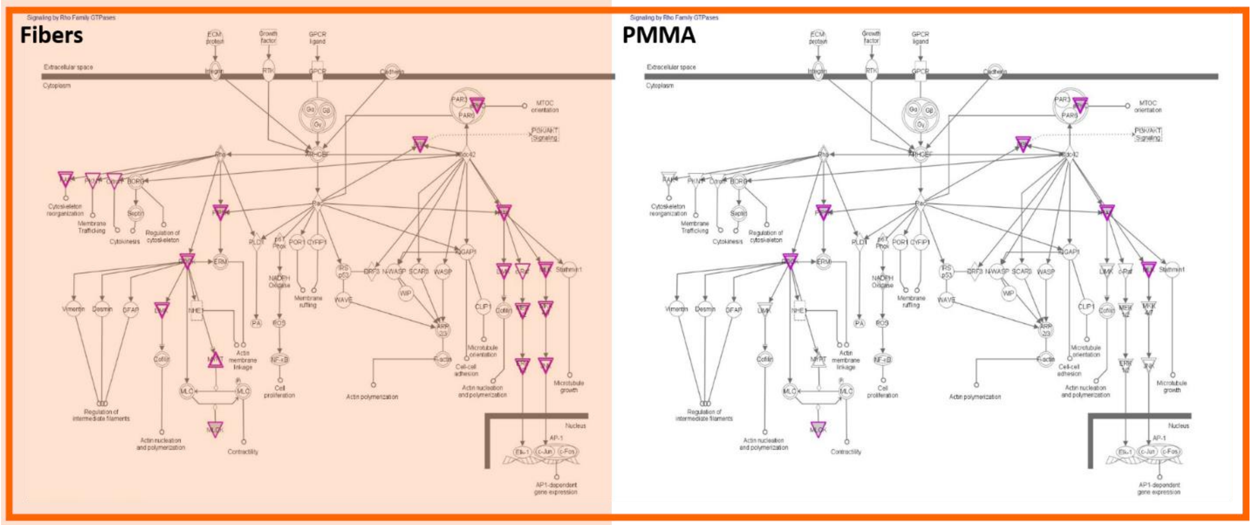

Fig S5. Shape parameters that were assessed using CellProfiler and the significance between fibrous and smooth topographies.

* Indicates significance compared to the untreated control of that topography type ($p<0.05$ by ANOVA with Fisher's test). Connecting bars indicate significance within an siRNA treatment (n=5 to 26, mean n= 11±4.3 cells)

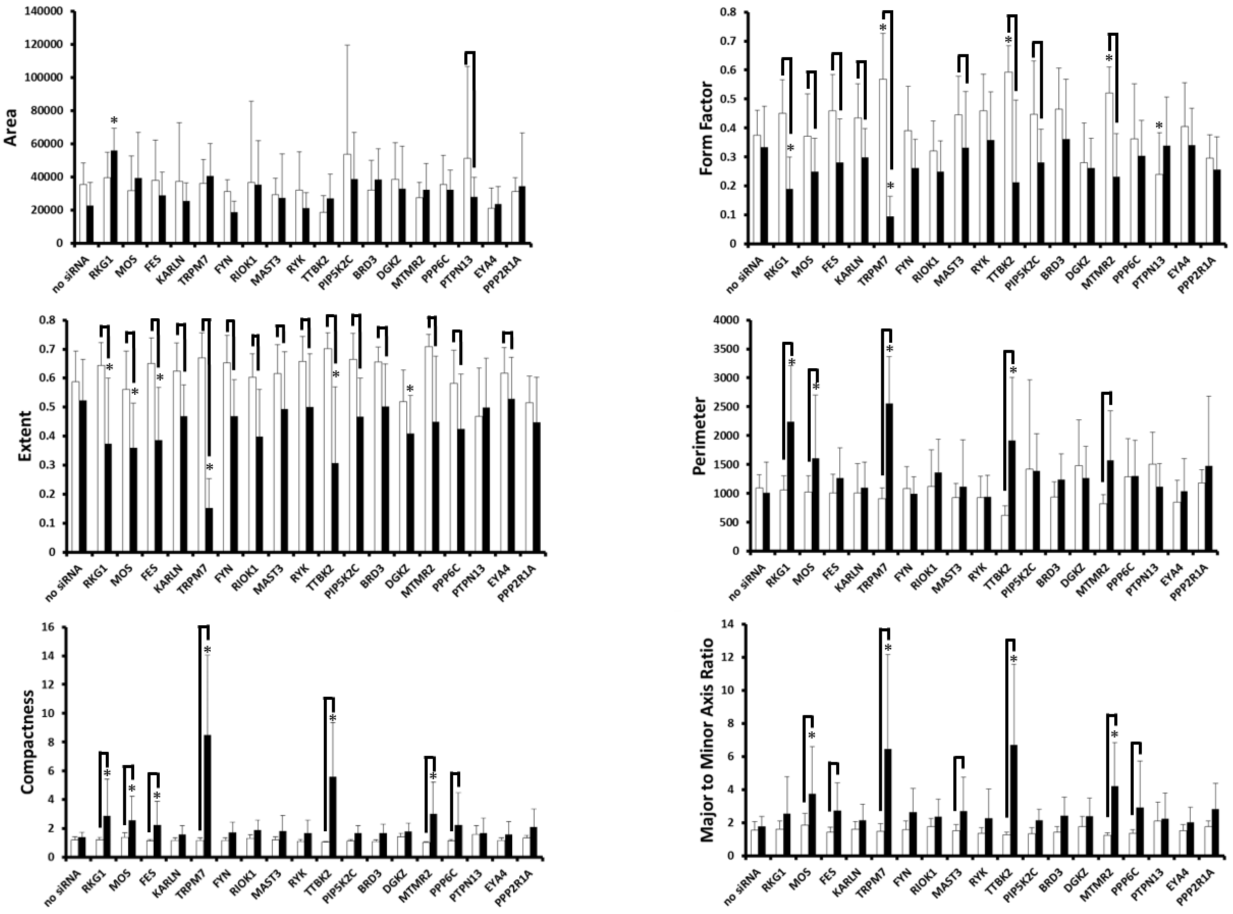

Supplementary File 1

	Kinase	
Gene_ID	Symbol	Definition
9451	EIF2AK3	eukaryotic translation initiation factor 2-alpha kinase 3
22848	AAK1	AP2 associated kinase 1
9625	AATK	apoptosis-associated tyrosine kinase
25	ABL1	v-abl Abelson murine leukemia viral oncogene homolog 1
27	ABL2	v-abl Abelson murine leukemia viral oncogene homolog 2 (arg, Abelson-related gene)
90	ACVR1	activin A receptor, type I
91	ACVR1B	activin A receptor, type IB
130399	ACVR1C	activin A receptor, type IC
92	ACVR2	activin A receptor, type II
93	ACVR2B	activin A receptor, type IIB
94	ACVRL1	activin A receptor type II-like 1
57143	ADCK1	aarF domain containing kinase 1
90956	ADCK2	aarF domain containing kinase 2
79934	ADCK4	aarF domain containing kinase 4
203054	ADCK5	aarF domain containing kinase 5
132	ADK	adenosine kinase
83440	ADPGK	ADP-dependent glucokinase
156	ADRBK1	adrenergic, beta, receptor kinase 1
157	ADRBK2	adrenergic, beta, receptor kinase 2
203	AK1	adenylate kinase 1
205	AK3	adenylate kinase 3
50808	AK3L1	adenylate kinase 3 like 1
26289	AK5	adenylate kinase 5
122481	AK7	adenylate kinase 7
207	AKT1	v-akt murine thymoma viral oncogene homolog 1
208	AKT2	v-akt murine thymoma viral oncogene homolog 2
10000	AKT3	v-akt murine thymoma viral oncogene homolog 3 (protein kinase B, gamma)
5832	ALDH18A1	aldehyde dehydrogenase 18 family, member A1
238	ALK	anaplastic lymphoma kinase (Ki-1)
80216	ALPK1	alpha-kinase 1
115701	ALPK2	alpha-kinase 2
57538	ALPK3	alpha-kinase 3
55437	ALS2CR2	amyotrophic lateral sclerosis 2 (juvenile) chromosome region, candidate 2
65061	ALS2CR7	amyotrophic lateral sclerosis 2 (juvenile) chromosome region, candidate 7
269	AMHR2	anti-Mullerian hormone receptor, type II
255239	ANKK1	ankyrin repeat and kinase domain containing 1
10290	APEG1	aortic preferentially expressed gene 1
369	ARAF	v-raf murine sarcoma 3611 viral oncogene homolog
9891	ARK5	AMP-activated protein kinase family member 5
472	ATM	ataxia telangiectasia mutated (includes complementation groups A, C and D)
545	ATR	ataxia telangiectasia and Rad3 related
9212	AURKB	aurora kinase B
6795	AURKC	aurora kinase C
558	AXL	AXL receptor tyrosine kinase
9223	BAIAP1	BAI1-associated protein 1
10295	BCKDK	branched chain ketoacid dehydrogenase kinase
613	BCR	breakpoint cluster region
640	BLK	B lymphoid tyrosine kinase
55589	BMP2K	BMP2 inducible kinase
657	BMPR1A	bone morphogenetic protein receptor, type IA
658	BMPR1B	bone morphogenetic protein receptor, type IB
659	BMPR2	bone morphogenetic protein receptor, type II (serine/threonine kinase)
660	BMX	BMX non-receptor tyrosine kinase
673	BRAF	v-raf murine sarcoma viral oncogene homolog B1
6046	BRD2	bromodomain containing 2
8019	BRD3	bromodomain containing 3
23476	BRD4	bromodomain containing 4
676	BRDT	bromodomain, testis-specific
84446	BRSK1	BR serine/threonine kinase 1
9024	BRSK2	BR serine/threonine kinase 2
695	BTK	Bruton agammaglobulinemia tyrosine kinase
699	BUB1	BUB1 budding uninhibited by benzimidazoles 1 homolog (yeast)
701	BUB1B	BUB1 budding uninhibited by benzimidazoles 1 homolog beta (yeast)
169436	C9orf96	chromosome 9 open reading frame 96
56997	CABC1	chaperone, ABC1 activity of bc1 complex like (S. pombe)
801	CALM1	calmodulin 1 (phosphorylase kinase, delta)
805	CALM2	calmodulin 2 (phosphorylase kinase, delta)
808	CALM3	calmodulin 3 (phosphorylase kinase, delta)
8536	CAMK1	calcium/calmodulin-dependent protein kinase I
57118	CAMK1D	calcium/calmodulin-dependent protein kinase ID
57172	CAMK1G	calcium/calmodulin-dependent protein kinase IG
815	CAMK2A	calcium/calmodulin-dependent protein kinase (CaM kinase) II alpha
816	CAMK2B	calcium/calmodulin-dependent protein kinase (CaM kinase) II beta
817	CAMK2D	calcium/calmodulin-dependent protein kinase (CaM kinase) II delta
818	CAMK2G	calcium/calmodulin-dependent protein kinase (CaM kinase) II gamma
814	CAMK4	calcium/calmodulin-dependent protein kinase IV
84254	CAMKK1	calcium/calmodulin-dependent protein kinase kinase 1, alpha
10645	CAMKK2	calcium/calmodulin-dependent protein kinase kinase 2, beta
79012	CAMKV	CaM kinase-like vesicle-associated
8573	CASK	calcium/calmodulin-dependent serine protein kinase (MAGUK family)
6347	CCL2	chemokine (C-C motif) ligand 2

23552	CCRK	cell cycle related kinase
983	CDC2	cell division cycle 2, G1 to S and G2 to M
984	CDC2L1	cell division cycle 2-like 1 (PITSLRE proteins)
8621	CDC2L5	cell division cycle 2-like 5 (cholinesterase-related cell division controller)
23097	CDC2L6	cell division cycle 2-like 6 (CDK8-like)
8476	CDC42BPA	CDC42 binding protein kinase alpha (DMPK-like)
9578	CDC42BPB	CDC42 binding protein kinase beta (DMPK-like)
55561	CDC42BPG	CDC42 binding protein kinase gamma (DMPK-like)
8317	CDC7	CDC7 cell division cycle 7 (S. cerevisiae)
8558	CDK10	cyclin-dependent kinase (CDC2-like) 10
1017	CDK2	cyclin-dependent kinase 2
1018	CDK3	cyclin-dependent kinase 3
1019	CDK4	cyclin-dependent kinase 4
1020	CDK5	cyclin-dependent kinase 5
1021	CDK6	cyclin-dependent kinase 6
1022	CDK7	cyclin-dependent kinase 7 (MO15 homolog, Xenopus laevis, cdk-activating kinase)
1024	CDK8	cyclin-dependent kinase 8
1025	CDK9	cyclin-dependent kinase 9 (CDC2-related kinase)
8814	CDKL1	cyclin-dependent kinase-like 1 (CDC2-related kinase)
8999	CDKL2	cyclin-dependent kinase-like 2 (CDC2-related kinase)
51265	CDKL3	cyclin-dependent kinase-like 3
344387	CDKL4	cyclin-dependent kinase-like 4
6792	CDKL5	cyclin-dependent kinase-like 5
64781	CERK	ceramide kinase
1111	CHEK1	CHK1 checkpoint homolog (S. pombe)
11200	CHEK2	CHK2 checkpoint homolog (S. pombe)
1119	CHKA	choline kinase alpha
1147	CHUK	conserved helix-loop-helix ubiquitous kinase
11113	CIT	citron (rho-interacting, serine/threonine kinase 21)
1158	CKM	creatine kinase, muscle
1159	CKMT1	creatine kinase, mitochondrial 1 (ubiquitous)
1160	CKMT2	creatine kinase, mitochondrial 2 (sarcomeric)
1195	CLK1	CDC-like kinase 1
1196	CLK2	CDC-like kinase 2
1198	CLK3	CDC-like kinase 3
57396	CLK4	CDC-like kinase 4
51727	CMPK	UMP-CMP kinase
80347	COASY	Coenzyme A synthase
10087	COL4A3BP	collagen, type IV, alpha 3 (Goodpasture antigen) binding protein
51755	CRK7	CDC2-related protein kinase 7
1399	CRKL	v-crk sarcoma virus CT10 oncogene homolog (avian)-like
1436	CSF1R	colony stimulating factor 1 receptor, formerly McDonough feline sarcoma viral (v-fms) oncogene homolog
1445	CSK	c-src tyrosine kinase
1452	CSNK1A1	casein kinase 1, alpha 1
122011	CSNK1A1L	casein kinase 1, alpha 1-like
1453	CSNK1D	casein kinase 1, delta
1454	CSNK1E	casein kinase 1, epsilon
53944	CSNK1G1	casein kinase 1, gamma 1
1455	CSNK1G2	casein kinase 1, gamma 2
1456	CSNK1G3	casein kinase 1, gamma 3
1457	CSNK2A1	casein kinase 2, alpha 1 polypeptide
1459	CSNK2A2	casein kinase 2, alpha prime polypeptide
1612	DAPK1	death-associated protein kinase 1
23604	DAPK2	death-associated protein kinase 2
1613	DAPK3	death-associated protein kinase 3
9201	DCAMKL1	doublecortin and CaM kinase-like 1
166614	DCAMKL2	doublecortin and CaM kinase-like 2
85443	DCAMKL3	doublecortin and CaM kinase-like 3
780	DDR1	discoidin domain receptor family, member 1
4921	DDR2	discoidin domain receptor family, member 2
1606	DGKA	diacylglycerol kinase, alpha 80kDa
1607	DGKB	diacylglycerol kinase, beta 90kDa
8527	DGKD	diacylglycerol kinase, delta 130kDa
8526	DGKE	diacylglycerol kinase, epsilon 64kDa
1608	DGKG	diacylglycerol kinase, gamma 90kDa
160851	DGKH	diacylglycerol kinase, eta
9162	DGKI	diacylglycerol kinase, iota
1609	DGKQ	diacylglycerol kinase, theta 110kDa
8525	DGKZ	diacylglycerol kinase, zeta 104kDa
1716	DGUOK	deoxyguanosine kinase
1739	DLG1	discs, large homolog 1 (Drosophila)
1740	DLG2	discs, large homolog 2, chapsyn-110 (Drosophila)
1741	DLG3	discs, large homolog 3 (neuroendocrine-dlg, Drosophila)
1742	DLG4	discs, large homolog 4 (Drosophila)
1760	DMPK	dystrophia myotonica-protein kinase
1841	DTYMK	deoxythymidylate kinase (thymidylate kinase)
1859	DYRK1A	dual-specificity tyrosine-(Y)-phosphorylation regulated kinase 1A
9149	DYRK1B	dual-specificity tyrosine-(Y)-phosphorylation regulated kinase 1B
8445	DYRK2	dual-specificity tyrosine-(Y)-phosphorylation regulated kinase 2
8444	DYRK3	dual-specificity tyrosine-(Y)-phosphorylation regulated kinase 3
8798	DYRK4	dual-specificity tyrosine-(Y)-phosphorylation regulated kinase 4
29904	EEF2K	eukaryotic elongation factor-2 kinase
1956	EGFR	epidermal growth factor receptor (erythroblastic leukemia viral (v-erb-b) oncogene homolog, avian)

27102	EIF2AK1	eukaryotic translation initiation factor 2-alpha kinase 1
5610	EIF2AK2	eukaryotic translation initiation factor 2-alpha kinase 2
440275	EIF2AK4	similar to GCN2 eIF2alpha kinase
2041	EPHA1	EPH receptor A1
284656	EPHA10	EPH receptor A10
1969	EPHA2	EPH receptor A2
2042	EPHA3	EPH receptor A3
2043	EPHA4	EPH receptor A4
2044	EPHA5	EPH receptor A5
2045	EPHA7	EPH receptor A7
2046	EPHA8	EPH receptor A8
2047	EPHB1	EPH receptor B1
2048	EPHB2	EPH receptor B2
2049	EPHB3	EPH receptor B3
2050	EPHB4	EPH receptor B4
2051	EPHB6	EPH receptor B6
2064	ERBB2	v-erb-b2 erythroblastic leukemia viral oncogene homolog 2, neuro/glioblastoma derived oncogene homolog (avian)
2065	ERBB3	v-erb-b2 erythroblastic leukemia viral oncogene homolog 3 (avian)
2066	ERBB4	v-erb-a erythroblastic leukemia viral oncogene homolog 4 (avian)
2081	ERN1	endoplasmic reticulum to nucleus signalling 1
10595	ERN2	endoplasmic reticulum to nucleus signalling 2
55500	ETNK1	ethanolamine kinase 1
55224	ETNK2	ethanolamine kinase 2
5394	EXOSC10	exosome component 10
2241	FER	fer (fps/fes related) tyrosine kinase (phosphoprotein NCP94)
2242	FES	feline sarcoma oncogene
2263	FGFR2	fibroblast growth factor receptor 2 (bacteria-expressed kinase, keratinocyte growth factor receptor, craniofacial dysostos
2261	FGFR3	fibroblast growth factor receptor 3 (achondroplasia, thanatophoric dwarfism)
2264	FGFR4	fibroblast growth factor receptor 4
53834	FGFRL1	fibroblast growth factor receptor-like 1
2268	FGR	Gardner-Rasheed feline sarcoma viral (v-fgr) oncogene homolog
124923	FLJ25006	hypothetical protein FLJ25006
152110	FLJ32685	hypothetical protein FLJ32685
2321	FLT1	fms-related tyrosine kinase 1 (vascular endothelial growth factor/vascular permeability factor receptor)
2322	FLT3	fms-related tyrosine kinase 3
2324	FLT4	fms-related tyrosine kinase 4
64122	FN3K	fructosamine 3 kinase
2475	FRAP1	FK506 binding protein 12-rapamycin associated protein 1
2444	FRK	fyn-related kinase
197258	FUK	fucokinase
2395	FXN	frataxin
2534	FYN	FYN oncogene related to SRC, FGR, YES
2580	GAK	cyclin G associated kinase
2585	GALK2	galactokinase 2
2645	GCK	glucokinase (hexokinase 4, maturity onset diabetes of the young 2)
2712	GK2	glycerol kinase 2
10020	GNE	glucosamine (UDP-N-acetyl)-2-epimerase/N-acetylmannosamine kinase
6011	GRK1	G protein-coupled receptor kinase 1
2868	GRK4	G protein-coupled receptor kinase 4
2869	GRK5	G protein-coupled receptor kinase 5
2870	GRK6	G protein-coupled receptor kinase 6
131890	GRK7	G protein-coupled receptor kinase 7
83903	GSG2	germ cell associated 2 (haspin)
2931	GSK3A	glycogen synthase kinase 3 alpha
2932	GSK3B	glycogen synthase kinase 3 beta
2984	GUCY2C	guanylate cyclase 2C (heat stable enterotoxin receptor)
3000	GUCY2D	guanylate cyclase 2D, membrane (retina-specific)
2986	GUCY2F	guanylate cyclase 2F, retinal
3055	HCK	hemopoietic cell kinase
204851	HIPK1	homeodomain interacting protein kinase 1
28996	HIPK2	homeodomain interacting protein kinase 2
10114	HIPK3	homeodomain interacting protein kinase 3
147746	HIPK4	homeodomain interacting protein kinase 4
3098	HK1	hexokinase 1
3099	HK2	hexokinase 2
3101	HK3	hexokinase 3 (white cell)
26353	HSPB8	heat shock 22kDa protein 8
30811	HUNK	hormonally upregulated Neu-associated kinase
22858	ICK	intestinal cell (MAK-like) kinase
3480	IGF1R	insulin-like growth factor 1 receptor
3482	IGF2R	insulin-like growth factor 2 receptor
9807	IHPK1	inositol hexaphosphate kinase 1
117283	IHPK3	inositol hexaphosphate kinase 3
3551	IKBKB	inhibitor of kappa light polypeptide gene enhancer in B-cells, kinase beta
9641	IKBKE	inhibitor of kappa light polypeptide gene enhancer in B-cells, kinase epsilon
3611	ILK	integrin-linked kinase
3643	INSR	insulin receptor
3645	INSRR	insulin receptor-related receptor
253430	IPMK	inositol polyphosphate multikinase
3654	IRAK1	interleukin-1 receptor-associated kinase 1
3656	IRAK2	interleukin-1 receptor-associated kinase 2
11213	IRAK3	interleukin-1 receptor-associated kinase 3
51135	IRAK4	interleukin-1 receptor-associated kinase 4

3702	ITK	IL2-inducible T-cell kinase
3705	ITPK1	inositol 1,3,4-triphosphate 5/6 kinase
3706	ITPKA	inositol 1,4,5-trisphosphate 3-kinase A
3707	ITPKB	inositol 1,4,5-trisphosphate 3-kinase B
80271	ITPKC	inositol 1,4,5-trisphosphate 3-kinase C
3716	JAK1	Janus kinase 1 (a protein tyrosine kinase)
3717	JAK2	Janus kinase 2 (a protein tyrosine kinase)
3718	JAK3	Janus kinase 3 (a protein tyrosine kinase, leukocyte)
11139	KALRN	kalirin, RhoGEF kin
3791	KDR	kinase insert domain receptor (a type III receptor tyrosine kinase)
3795	KHK	ketohexokinase (fructokinase)
23387	KIAA0999	KIAA0999 protein
84451	KIAA1804	mixed lineage kinase 4
3815	KIT	v-kit Hardy-Zuckerman 4 feline sarcoma viral oncogene homolog
8844	KSR	kinase suppressor of ras
283455	KSR2	kinase suppressor of ras 2
9113	LATS1	LATS, large tumor suppressor, homolog 1 (Drosophila)
26524	LATS2	LATS, large tumor suppressor, homolog 2 (Drosophila)
3932	LCK	lymphocyte-specific protein tyrosine kinase
3984	LIMK1	LIM domain kinase 1
3985	LIMK2	LIM domain kinase 2
22853	LMTK2	lemur tyrosine kinase 2
114783	LMTK3	lemur tyrosine kinase 3
79705	LRRK1	leucine-rich repeat kinase 1
120892	LRRK2	leucine-rich repeat kinase 2
4058	LTK	leukocyte tyrosine kinase
92335	LYK5	protein kinase LYK5
4067	LYN	v-yes-1 Yamaguchi sarcoma viral related oncogene homolog
260425	MAGI3	membrane-associated guanylate kinase-related (MAGI-3)
4117	MAK	male germ cell-associated kinase
5604	MAP2K1	mitogen-activated protein kinase kinase 1
5605	MAP2K2	mitogen-activated protein kinase kinase 2
5606	MAP2K3	mitogen-activated protein kinase kinase 3
6416	MAP2K4	mitogen-activated protein kinase kinase 4
5607	MAP2K5	mitogen-activated protein kinase kinase 5
5608	MAP2K6	mitogen-activated protein kinase kinase 6
5609	MAP2K7	mitogen-activated protein kinase kinase 7
4214	MAP3K1	mitogen-activated protein kinase kinase kinase 1
4294	MAP3K10	mitogen-activated protein kinase kinase kinase 10
4296	MAP3K11	mitogen-activated protein kinase kinase kinase 11
7786	MAP3K12	mitogen-activated protein kinase kinase kinase 12
9175	MAP3K13	mitogen-activated protein kinase kinase kinase 13
9020	MAP3K14	mitogen-activated protein kinase kinase kinase 14
389840	MAP3K15	mitogen-activated protein kinase kinase kinase 15
10746	MAP3K2	mitogen-activated protein kinase kinase kinase 2
4215	MAP3K3	mitogen-activated protein kinase kinase kinase 3
4216	MAP3K4	mitogen-activated protein kinase kinase kinase 4
4217	MAP3K5	mitogen-activated protein kinase kinase kinase 5
9064	MAP3K6	mitogen-activated protein kinase kinase kinase 6
6885	MAP3K7	mitogen-activated protein kinase kinase kinase 7
1326	MAP3K8	mitogen-activated protein kinase kinase kinase 8
4293	MAP3K9	mitogen-activated protein kinase kinase kinase 9
11184	MAP4K1	mitogen-activated protein kinase kinase kinase kinase 1
5871	MAP4K2	mitogen-activated protein kinase kinase kinase kinase 2
8491	MAP4K3	mitogen-activated protein kinase kinase kinase kinase 3
9448	MAP4K4	mitogen-activated protein kinase kinase kinase kinase 4
11183	MAP4K5	mitogen-activated protein kinase kinase kinase kinase 5
5594	MAPK1	mitogen-activated protein kinase 1
5602	MAPK10	mitogen-activated protein kinase 10
5600	MAPK11	mitogen-activated protein kinase 11
6300	MAPK12	mitogen-activated protein kinase 12
5603	MAPK13	mitogen-activated protein kinase 13
1432	MAPK14	mitogen-activated protein kinase 14
5595	MAPK3	mitogen-activated protein kinase 3
5596	MAPK4	mitogen-activated protein kinase 4
5597	MAPK6	mitogen-activated protein kinase 6
5598	MAPK7	mitogen-activated protein kinase 7
5599	MAPK8	mitogen-activated protein kinase 8
5601	MAPK9	mitogen-activated protein kinase 9
9261	MAPKAPK2	mitogen-activated protein kinase-activated protein kinase 2
7867	MAPKAPK3	mitogen-activated protein kinase-activated protein kinase 3
8550	MAPKAPK5	mitogen-activated protein kinase-activated protein kinase 5
4139	MARK1	MAP/microtubule affinity-regulating kinase 1
2011	MARK2	MAP/microtubule affinity-regulating kinase 2
4140	MARK3	MAP/microtubule affinity-regulating kinase 3
57787	MARK4	MAP/microtubule affinity-regulating kinase 4
51765	MASK	Mst3 and SOK1-related kinase
22983	MAST1	microtubule associated serine/threonine kinase 1
23139	MAST2	microtubule associated serine/threonine kinase 2
23031	MAST3	microtubule associated serine/threonine kinase 3
23227	MAST4	microtubule associated serine/threonine kinase family member 4
84930	MASTL	microtubule associated serine/threonine kinase-like
4145	MATK	megakaryocyte-associated tyrosine kinase

9833	MELK	maternal embryonic leucine zipper kinase
10461	MERTK	c-mer proto-oncogene tyrosine kinase
4233	MET	met proto-oncogene (hepatocyte growth factor receptor)
93627	MGC16169	hypothetical protein MGC16169
167359	MGC42105	hypothetical protein MGC42105
50488	MINK1	misshapen-like kinase 1 (zebrafish)
8569	MKNK1	MAP kinase interacting serine/threonine kinase 1
2872	MKNK2	MAP kinase interacting serine/threonine kinase 2
91807	MLCK	cardiac-MyBP-C associated Ca/CaM kinase
197259	MLKL	mixed lineage kinase domain-like
4342	MOS	v-mos Moloney murine sarcoma viral oncogene homolog
4354	MPP1	membrane protein, palmitoylated 1, 55kDa
4355	MPP2	membrane protein, palmitoylated 2 (MAGUK p55 subfamily member 2)
4356	MPP3	membrane protein, palmitoylated 3 (MAGUK p55 subfamily member 3)
4486	MST1R	macrophage stimulating 1 receptor (c-met-related tyrosine kinase)
55750	MULK	multiple substrate lipid kinase
4593	MUSK	muscle, skeletal, receptor tyrosine kinase
4598	MVK	mevalonate kinase (mevalonic aciduria)
4638	MYLK	myosin, light polypeptide kinase
85366	MYLK2	myosin light chain kinase 2, skeletal muscle
53904	MYO3A	myosin IIIA
140469	MYO3B	myosin IIIB
65220	NADK	NAD kinase
55577	NAGK	N-acetylglucosamine kinase
162417	NAGS	N-acetylglutamate synthase
4750	NEK1	NIMA (never in mitosis gene a)-related kinase 1
79858	NEK11	NIMA (never in mitosis gene a)- related kinase 11
4751	NEK2	NIMA (never in mitosis gene a)-related kinase 2
4752	NEK3	NIMA (never in mitosis gene a)-related kinase 3
6787	NEK4	NIMA (never in mitosis gene a)-related kinase 4
341676	NEK5	similar to Serine/threonine-protein kinase Nek1 (NimA-related protein kinase 1)
10783	NEK6	NIMA (never in mitosis gene a)-related kinase 6
140609	NEK7	NIMA (never in mitosis gene a)-related kinase 7
284086	NEK8	NIMA (never in mitosis gene a)- related kinase 8
91754	NEK9	NIMA (never in mitosis gene a)- related kinase 9
51701	NLK	nemo like kinase
4831	NME2	non-metastatic cells 2, protein (NM23B) expressed in
8382	NME5	non-metastatic cells 5, protein expressed in (nucleoside-diphosphate kinase)
10201	NME6	non-metastatic cells 6, protein expressed in (nucleoside-diphosphate kinase)
29922	NME7	non-metastatic cells 7, protein expressed in (nucleoside-diphosphate kinase)
4881	NPR1	natriuretic peptide receptor A/guanylate cyclase A (atrionatriuretic peptide receptor A)
4882	NPR2	natriuretic peptide receptor B/guanylate cyclase B (atrionatriuretic peptide receptor B)
29959	NRBP	nuclear receptor binding protein
340371	NRBP2	nuclear receptor binding protein 2
203447	NRK	Nik related kinase
4914	NTRK1	neurotrophic tyrosine kinase, receptor, type 1
4915	NTRK2	neurotrophic tyrosine kinase, receptor, type 2
4916	NTRK3	neurotrophic tyrosine kinase, receptor, type 3
9943	OXSR1	oxidative-stress responsive 1
57147	PACE-1	ezrin-binding partner PACE-1
5058	PAK1	p21/Cdc42/Rac1-activated kinase 1 (STE20 homolog, yeast)
5062	PAK2	p21 (CDKN1A)-activated kinase 2
5063	PAK3	p21 (CDKN1A)-activated kinase 3
10298	PAK4	p21(CDKN1A)-activated kinase 4
56924	PAK6	p21(CDKN1A)-activated kinase 6
57144	PAK7	p21(CDKN1A)-activated kinase 7
53354	PANK1	pantothenate kinase 1
80025	PANK2	pantothenate kinase 2 (Hallervorden-Spatz syndrome)
79646	PANK3	pantothenate kinase 3
55229	PANK4	pantothenate kinase 4
23178	PASK	PAS domain containing serine/threonine kinase
55872	PBK	PDZ binding kinase
5127	PCTK1	PCTAIRE protein kinase 1
5128	PCTK2	PCTAIRE protein kinase 2
5129	PCTK3	PCTAIRE protein kinase 3
5156	PDGFRA	platelet-derived growth factor receptor, alpha polypeptide
5159	PDGFRB	platelet-derived growth factor receptor, beta polypeptide
5157	PDGFRL	platelet-derived growth factor receptor-like
149420	PDIK1L	PDLIM1 interacting kinase 1 like
5163	PDK1	pyruvate dehydrogenase kinase, isoenzyme 1
5164	PDK2	pyruvate dehydrogenase kinase, isoenzyme 2
5165	PDK3	pyruvate dehydrogenase kinase, isoenzyme 3
5166	PDK4	pyruvate dehydrogenase kinase, isoenzyme 4
5170	PDPK1	3-phosphoinositide dependent protein kinase-1
8566	PDXK	pyridoxal (pyridoxine, vitamin B6) kinase
5207	PFKFB1	6-phosphofructo-2-kinase/fructose-2,6-biphosphatase 1
5208	PFKFB2	6-phosphofructo-2-kinase/fructose-2,6-biphosphatase 2
5209	PFKFB3	6-phosphofructo-2-kinase/fructose-2,6-biphosphatase 3
5210	PFKFB4	6-phosphofructo-2-kinase/fructose-2,6-biphosphatase 4
5211	PFKL	phosphofructokinase, liver
5213	PFKM	phosphofructokinase, muscle
5214	PFKP	phosphofructokinase, platelet
5218	PFTK1	PFTAIRE protein kinase 1

5230	PGK1	phosphoglycerate kinase 1
5232	PGK2	phosphoglycerate kinase 2
5255	PHKA1	phosphorylase kinase, alpha 1 (muscle)
5256	PHKA2	phosphorylase kinase, alpha 2 (liver)
5257	PHKB	phosphorylase kinase, beta
5260	PHKG1	phosphorylase kinase, gamma 1 (muscle)
5261	PHKG2	phosphorylase kinase, gamma 2 (testis)
55300	PI4K2B	phosphatidylinositol 4-kinase type-II beta
55361	PI4KII	phosphatidylinositol 4-kinase type II
5286	PIK3C2A	phosphoinositide-3-kinase, class 2, alpha polypeptide
5287	PIK3C2B	phosphoinositide-3-kinase, class 2, beta polypeptide
5288	PIK3C2G	phosphoinositide-3-kinase, class 2, gamma polypeptide
5289	PIK3C3	phosphoinositide-3-kinase, class 3
5290	PIK3CA	phosphoinositide-3-kinase, catalytic, alpha polypeptide
5291	PIK3CB	phosphoinositide-3-kinase, catalytic, beta polypeptide
5293	PIK3CD	phosphoinositide-3-kinase, catalytic, delta polypeptide
5294	PIK3CG	phosphoinositide-3-kinase, catalytic, gamma polypeptide
30849	PIK3R4	phosphoinositide-3-kinase, regulatory subunit 4, p150
5297	PIK4CA	phosphatidylinositol 4-kinase, catalytic, alpha polypeptide
5298	PIK4CB	phosphatidylinositol 4-kinase, catalytic, beta polypeptide
5292	PIM1	pim-1 oncogene
11040	PIM2	pim-2 oncogene
415116	PIM3	pim-3 oncogene
65018	PINK1	PTEN induced putative kinase 1
8395	PIP5K1B	phosphatidylinositol-4-phosphate 5-kinase, type I, beta
23396	PIP5K1C	phosphatidylinositol-4-phosphate 5-kinase, type I, gamma
5305	PIP5K2A	phosphatidylinositol-4-phosphate 5-kinase, type II, alpha
8396	PIP5K2B	phosphatidylinositol-4-phosphate 5-kinase, type II, beta
79837	PIP5K2C	phosphatidylinositol-4-phosphate 5-kinase, type II, gamma
200576	PIP5K3	phosphatidylinositol-3-phosphate/phosphatidylinositol 5-kinase, type III
138429	PIP5KL1	phosphatidylinositol-4-phosphate 5-kinase-like 1
5313	PKLR	pyruvate kinase, liver and RBC
5315	PKM2	pyruvate kinase, muscle
9088	PKMYT1	protein kinase, membrane associated tyrosine/threonine 1
5585	PKN1	protein kinase N1
5586	PKN2	protein kinase N2
29941	PKN3	protein kinase N3
5347	PLK1	polo-like kinase 1 (Drosophila)
10769	PLK2	polo-like kinase 2 (Drosophila)
1263	PLK3	polo-like kinase 3 (Drosophila)
10733	PLK4	polo-like kinase 4 (Drosophila)
10654	PMVK	phosphomevalonate kinase
5562	PRKAA1	protein kinase, AMP-activated, alpha 1 catalytic subunit
5563	PRKAA2	protein kinase, AMP-activated, alpha 2 catalytic subunit
5566	PRKACA	protein kinase, cAMP-dependent, catalytic, alpha
5567	PRKACB	protein kinase, cAMP-dependent, catalytic, beta
5568	PRKACG	protein kinase, cAMP-dependent, catalytic, gamma
5578	PRKCA	protein kinase C, alpha
5579	PRKCB1	protein kinase C, beta 1
5580	PRKCD	protein kinase C, delta
5581	PRKCE	protein kinase C, epsilon
5582	PRKCG	protein kinase C, gamma
5583	PRKCH	protein kinase C, eta
5584	PRKCI	protein kinase C, iota
5588	PRKCQ	protein kinase C, theta
5590	PRKCZ	protein kinase C, zeta
5587	PRKD1	protein kinase D1
25865	PRKD2	protein kinase D2
23683	PRKD3	protein kinase D3
5591	PRKDC	protein kinase, DNA-activated, catalytic polypeptide
5592	PRKG1	protein kinase, cGMP-dependent, type I
5593	PRKG2	protein kinase, cGMP-dependent, type II
5613	PRKX	protein kinase, X-linked
5616	PRKY	protein kinase, Y-linked
8899	PRPF4B	PRP4 pre-mRNA processing factor 4 homolog B (yeast)
5631	PRPS1	phosphoribosyl pyrophosphate synthetase 1
5634	PRPS2	phosphoribosyl pyrophosphate synthetase 2
5681	PSKH1	protein serine kinase H1
85481	PSKH2	protein serine kinase H2
5747	PTK2	PTK2 protein tyrosine kinase 2
2185	PTK2B	PTK2B protein tyrosine kinase 2 beta
5753	PTK6	PTK6 protein tyrosine kinase 6
5754	PTK7	PTK7 protein tyrosine kinase 7
5756	PTK9	PTK9 protein tyrosine kinase 9
11344	PTK9L	PTK9L protein tyrosine kinase 9-like (A6-related protein)
54899	PXK	PX domain containing serine/threonine kinase
5894	RAF1	v-raf-1 murine leukemia viral oncogene homolog 1
5891	RAGE	renal tumor antigen
64080	RBKS	ribokinase
5979	RET	ret proto-oncogene (multiple endocrine neoplasia and medullary thyroid carcinoma 1, Hirschsprung disease)
55312	RFK	riboflavin kinase
5987	RFP	ret finger protein
83732	RIOK1	RIO kinase 1 (yeast)

55781	RIOK2	RIO kinase 2 (yeast)
8780	RIOK3	RIO kinase 3 (yeast)
8737	RIPK1	receptor (TNFRSF)-interacting serine-threonine kinase 1
8767	RIPK2	receptor-interacting serine-threonine kinase 2
11035	RIPK3	receptor-interacting serine-threonine kinase 3
54101	RIPK4	receptor-interacting serine-threonine kinase 4
25778	RIPK5	receptor interacting protein kinase 5
6041	RNASEL	ribonuclease L (2',5'-oligoisoadenylate synthetase-dependent)
6093	ROCK1	Rho-associated, coiled-coil containing protein kinase 1
9475	ROCK2	Rho-associated, coiled-coil containing protein kinase 2
4919	ROR1	receptor tyrosine kinase-like orphan receptor 1
4920	ROR2	receptor tyrosine kinase-like orphan receptor 2
6098	ROS1	v-ros UR2 sarcoma virus oncogene homolog 1 (avian)
6195	RPS6KA1	ribosomal protein S6 kinase, 90kDa, polypeptide 1
6196	RPS6KA2	ribosomal protein S6 kinase, 90kDa, polypeptide 2
6197	RPS6KA3	ribosomal protein S6 kinase, 90kDa, polypeptide 3
8986	RPS6KA4	ribosomal protein S6 kinase, 90kDa, polypeptide 4
9252	RPS6KA5	ribosomal protein S6 kinase, 90kDa, polypeptide 5
27330	RPS6KA6	ribosomal protein S6 kinase, 90kDa, polypeptide 6
6198	RPS6KB1	ribosomal protein S6 kinase, 70kDa, polypeptide 1
6199	RPS6KB2	ribosomal protein S6 kinase, 70kDa, polypeptide 2
26750	RPS6KC1	ribosomal protein S6 kinase, 52kDa, polypeptide 1
83694	RPS6KL1	ribosomal protein S6 kinase-like 1
6259	RYK	RYK receptor-like tyrosine kinase
388228	SBK1	SH3-binding domain kinase 1
8631	SCAP1	src family associated phosphoprotein 1
8935	SCAP2	src family associated phosphoprotein 2
57410	SCYL1	SCY1-like 1 (S. cerevisiae)
55681	SCYL2	SCY1-like 2 (S. cerevisiae)
22928	SEPHS2	selenophosphate synthetase 2
6446	SGK	serum/glucocorticoid regulated kinase
10110	SGK2	serum/glucocorticoid regulated kinase 2
23678	SGKL	serum/glucocorticoid regulated kinase-like
9748	SLK	STE20-like kinase (yeast)
23049	SMG1	PI-3-kinase-related kinase SMG-1
81788	SNARK	likely ortholog of rat SNF1/AMP-activated protein kinase
150094	SNF1LK	SNF1-like kinase
23235	SNF1LK2	SNF1-like kinase 2
54861	SNRK	SNF related kinase
8877	SPHK1	sphingosine kinase 1
56848	SPHK2	sphingosine kinase 2
6714	SRC	v-src sarcoma (Schmidt-Ruppin A-2) viral oncogene homolog (avian)
6725	SRMS	src-related kinase lacking C-terminal regulatory tyrosine and N-terminal myristylation sites
6732	SRPK1	SFRS protein kinase 1
6733	SRPK2	SFRS protein kinase 2
83983	SSTK	serine/threonine protein kinase SSTK
6793	STK10	serine/threonine kinase 10
6794	STK11	serine/threonine kinase 11 (Peutz-Jeghers syndrome)
8576	STK16	serine/threonine kinase 16
9263	STK17A	serine/threonine kinase 17a (apoptosis-inducing)
9262	STK17B	serine/threonine kinase 17b (apoptosis-inducing)
8859	STK19	serine/threonine kinase 19
81629	STK22C	serine/threonine kinase 22C (spermiogenesis associated)
26576	STK23	serine/threonine kinase 23
8428	STK24	serine/threonine kinase 24 (STE20 homolog, yeast)
10494	STK25	serine/threonine kinase 25 (STE20 homolog, yeast)
6788	STK3	serine/threonine kinase 3 (STE20 homolog, yeast)
56164	STK31	serine/threonine kinase 31
202374	STK32A	serine/threonine kinase 32A
55351	STK32B	serine/threonine kinase 32B
282974	STK32C	serine/threonine kinase 32C
65975	STK33	serine/threonine kinase 33
140901	STK35	serine/threonine kinase 35
27148	STK36	serine/threonine kinase 36 (fused homolog, Drosophila)
11329	STK38	serine/threonine kinase 38
23012	STK38L	serine/threonine kinase 38 like
27347	STK39	serine threonine kinase 39 (STE20/SPS1 homolog, yeast)
6789	STK4	serine/threonine kinase 4
83931	STK40	serine/threonine kinase 40
6790	STK6	serine/threonine kinase 6
55359	STYK1	serine/threonine/tyrosine kinase 1
6850	SYK	spleen tyrosine kinase
6872	TAF1	TAF1 RNA polymerase II, TATA box binding protein (TBP)-associated factor, 250kDa
57551	TAOK1	TAO kinase 1
9344	TAOK2	TAO kinase 2
51347	TAOK3	TAO kinase 3
29110	TBK1	TANK-binding kinase 1
7006	TEC	tec protein tyrosine kinase
7010	TEK	TEK tyrosine kinase, endothelial (venous malformations, multiple cutaneous and mucosal)
7016	TESK1	testis-specific kinase 1
10420	TESK2	testis-specific kinase 2
56155	TEX14	testis expressed sequence 14
7046	TGFBR1	transforming growth factor, beta receptor I (activin A receptor type II-like kinase, 53kDa)

7048	TGFBR2	transforming growth factor, beta receptor II (70/80kDa)
7075	TIE1	tyrosine kinase with immunoglobulin-like and EGF-like domains 1
9414	TJP2	tight junction protein 2 (zona occludens 2)
7084	TK2	thymidine kinase 2, mitochondrial
9874	TLK1	tousled-like kinase 1
11011	TLK2	tousled-like kinase 2
23043	TNIK	TRAF2 and NCK interacting kinase
8711	TNK1	tyrosine kinase, non-receptor, 1
10188	TNK2	tyrosine kinase, non-receptor, 2
51086	TNNI3K	TNNI3 interacting kinase
112858	TP53RK	TP53 regulating kinase
27010	TPK1	thiamin pyrophosphokinase 1
10221	TRIB1	tribbles homolog 1 (Drosophila)
28951	TRIB2	tribbles homolog 2 (Drosophila)
57761	TRIB3	tribbles homolog 3 (Drosophila)
10155	TRIM28	tripartite motif-containing 28
51592	TRIM33	tripartite motif-containing 33
7204	TRIO	triple functional domain (PTPRF interacting)
140803	TRPM6	transient receptor potential cation channel, subfamily M, member 6
54822	TRPM7	transient receptor potential cation channel, subfamily M, member 7
8295	TRRAP	transformation/transcription domain-associated protein
23617	TSSK2	testis-specific serine kinase 2
283629	TSSK4	testis-specific serine kinase 4
84630	TTBK1	tau tubulin kinase 1
146057	TTBK2	tau tubulin kinase 2
7272	TTK	TTK protein kinase
7273	TTN	titin
7294	TXK	TXK tyrosine kinase
7297	TYK2	tyrosine kinase 2
7301	TYRO3	TYRO3 protein tyrosine kinase
83549	UCK1	uridine-cytidine kinase 1
7371	UCK2	uridine-cytidine kinase 2
54963	UCKL1	uridine-cytidine kinase 1-like 1
127933	UHMK1	U2AF homology motif (UHM) kinase 1
8408	ULK1	unc-51-like kinase 1 (C. elegans)
9706	ULK2	unc-51-like kinase 2 (C. elegans)
25989	ULK3	unc-51-like kinase 3 (C. elegans)
54986	ULK4	unc-51-like kinase 4 (C. elegans)
7443	VRK1	vaccinia related kinase 1
7444	VRK2	vaccinia related kinase 2
51231	VRK3	vaccinia related kinase 3
7465	WEE1	WEE1 homolog (S. pombe)
65125	WNK1	WNK lysine deficient protein kinase 1
65268	WNK2	WNK lysine deficient protein kinase 2
65267	WNK3	WNK lysine deficient protein kinase 3
65266	WNK4	WNK lysine deficient protein kinase 4
9942	XYLB	xylulokinase homolog (H. influenzae)
7525	YES1	v-yes-1 Yamaguchi sarcoma viral oncogene homolog 1
80122	YSK4	Yeast Sps1/Ste20-related kinase 4 (S. cerevisiae)
51776	ZAK	sterile alpha motif and leucine zipper containing kinase AZK
7535	ZAP70	zeta-chain (TCR) associated protein kinase 70kDa
79834		KIAA2002 protein
91461		hypothetical protein BC007901
157285		hypothetical protein DKFZp761P0423
340156		hypothetical protein LOC340156
390975		similar to protein kinase Bsk146
84197		hypothetical protein FLJ23356
	**Phophatase**	
**Gene_ID**	**Symbol**	**Definition**
52	ACP1	acid phosphatase 1, soluble
53	ACP2	acid phosphatase 2, lysosomal
54	ACP5	acid phosphatase 5, tartrate resistant
51205	ACP6	acid phosphatase 6, lysophosphatidic
92370	ACPL2	acid phosphatase-like 2
55	ACPP	acid phosphatase, prostate
93650	ACPT	acid phosphatase, testicular
248	ALPI	alkaline phosphatase, intestinal
249	ALPL	alkaline phosphatase, liver/bone/kidney
250	ALPP	alkaline phosphatase, placental (Regan isozyme)
251	ALPPL2	alkaline phosphatase, placental-like 2
669	BPGM	2,3-bisphosphoglycerate mutase
10380	BPNT1	3'(2'), 5'-bisphosphate nucleotidase 1
818	CAMK2G	calcium/calmodulin-dependent protein kinase (CaM kinase) II gamma
8556	CDC14A	CDC14 cell division cycle 14 homolog A (S. cerevisiae)
8555	CDC14B	CDC14 cell division cycle 14 homolog B (S. cerevisiae)
168448	CDC14C	CDC14 cell division cycle 14 homolog C (S. cerevisiae)
993	CDC25A	cell division cycle 25A
994	CDC25B	cell division cycle 25B
995	CDC25C	cell division cycle 25C
1033	CDKN3	cyclin-dependent kinase inhibitor 3 (CDK2-associated dual specificity phosphatase)
8483	CILP	cartilage intermediate layer protein, nucleotide pyrophosphohydrolase
9150	CTDP1	CTD (carboxy-terminal domain, RNA polymerase II, polypeptide A) phosphatase, subunit 1
58190	CTDSP1	CTD (carboxy-terminal domain, RNA polymerase II, polypeptide A) small phosphatase 1

10106	CTDSP2	CTD (carboxy-terminal domain, RNA polymerase II, polypeptide A) small phosphatase 2
10217	CTDSPL	CTD (carboxy-terminal domain, RNA polymerase II, polypeptide A)small phosphatase-like
27071	DAPP1	dual adaptor of phosphotyrosine and 3-phosphoinositides
9829	DNAJC6	DnaJ (Hsp40) homolog, subfamily C, member 6
57171	DOLPP1	dolichyl pyrophosphate phosphatase 1
338599	DUPD1	dual specificity phosphatase and pro isomerase domain containing 1
1843	DUSP1	dual specificity phosphatase 1
11221	DUSP10	dual specificity phosphatase 10
8446	DUSP11	dual specificity phosphatase 11 (RNA/RNP complex 1-interacting)
11266	DUSP12	dual specificity phosphatase 12
51207	DUSP13	dual specificity phosphatase 13
11072	DUSP14	dual specificity phosphatase 14
128853	DUSP15	dual specificity phosphatase 15
80824	DUSP16	dual specificity phosphatase 16
150290	DUSP18	dual specificity phosphatase 18
142679	DUSP19	dual specificity phosphatase 19
1844	DUSP2	dual specificity phosphatase 2
63904	DUSP21	dual specificity phosphatase 21
56940	DUSP22	dual specificity phosphatase 22
54935	DUSP23	dual specificity phosphatase 23
78986	DUSP26	dual specificity phosphatase 26 (putative)
1845	DUSP3	dual specificity phosphatase 3 (vaccinia virus phosphatase VH1-related)
1846	DUSP4	dual specificity phosphatase 4
1847	DUSP5	dual specificity phosphatase 5
1848	DUSP6	dual specificity phosphatase 6
1849	DUSP7	dual specificity phosphatase 7
1850	DUSP8	dual specificity phosphatase 8
1852	DUSP9	dual specificity phosphatase 9
5167	ENPP1	ectonucleotide pyrophosphatase/phosphodiesterase 1
5168	ENPP2	ectonucleotide pyrophosphatase/phosphodiesterase 2 (autotaxin)
5169	ENPP3	ectonucleotide pyrophosphatase/phosphodiesterase 3
7957	EPM2A	epilepsy, progressive myoclonus type 2A, Lafora disease (laforin)
2138	EYA1	eyes absent homolog 1 (Drosophila)
2139	EYA2	eyes absent homolog 2 (Drosophila)
2140	EYA3	eyes absent homolog 3 (Drosophila)
2070	EYA4	eyes absent homolog 4 (Drosophila)
2203	FBP1	fructose-1,6-bisphosphatase 1
8789	FBP2	fructose-1,6-bisphosphatase 2
196515	FLJ30092	AF-1 specific protein phosphatase
2538	G6PC	glucose-6-phosphatase, catalytic (glycogen storage disease type I,von Gierke disease)
3612	IMPA1	inositol(myo)-1(or 4)-monophosphatase 1
3613	IMPA2	inositol(myo)-1(or 4)-monophosphatase 2
54928	IMPAD1	inositol monophosphatase domain containing 1
3628	INPP1	inositol polyphosphate-1-phosphatase
3631	INPP4A	inositol polyphosphate-4-phosphatase, type I, 107kDa
8821	INPP4B	inositol polyphosphate-4-phosphatase, type II, 105kDa
3632	INPP5A	inositol polyphosphate-5-phosphatase, 40kDa
3633	INPP5B	inositol polyphosphate-5-phosphatase, 75kDa
3635	INPP5D	inositol polyphosphate-5-phosphatase, 145kDa
56623	INPP5E	inositol polyphosphate-5-phosphatase, 72 kDa
22876	INPP5F	inositol polyphosphate-5-phosphatase F
3636	INPPL1	inositol polyphosphate phosphatase-like 1
3704	ITPA	inosine triphosphatase (nucleoside triphosphate pyrophosphatase)
55709	KBTBD4	kelch repeat and BTB (POZ) domain containing 4
27143	KIAA1274	KIAA1274
64077	LHPP	phospholysine phosphohistidine inorganic pyrophosphate phosphatase
9562	MINPP1	multiple inositol polyphosphate histidine phosphatase, 1
4534	MTM1	myotubularin 1
8776	MTMR1	myotubularin related protein 1
8898	MTMR2	myotubularin related protein 2
8897	MTMR3	myotubularin related protein 3
9110	MTMR4	myotubularin related protein 4
9107	MTMR6	myotubularin related protein 6
9108	MTMR7	myotubularin related protein 7
55613	MTMR8	myotubularin related protein 8
4952	OCRL	oculocerebrorenal syndrome of Lowe
57546	PDP2	pyruvate dehydrogenase phosphatase isoenzyme 2
57026	PDXP	pyridoxal (pyridoxine, vitamin B6) phosphatase
5207	PFKFB1	6-phosphofructo-2-kinase/fructose-2,6-biphosphatase 1
5208	PFKFB2	6-phosphofructo-2-kinase/fructose-2,6-biphosphatase 2
5209	PFKFB3	6-phosphofructo-2-kinase/fructose-2,6-biphosphatase 3
5210	PFKFB4	6-phosphofructo-2-kinase/fructose-2,6-biphosphatase 4
23035	PHLPPL	PH domain and leucine rich repeat protein phosphatase-like
162466	PHOSPHO1	phosphatase, orphan 1
29085	PHPT1	phosphohistidine phosphatase 1
27124	PIB5PA	phosphatidylinositol (4,5) bisphosphate 5-phosphatase, A
23239	PHLPP	PH domain and leucine rich repeat protein phosphatase
51400	PME	-1 protein phosphatase methylesterase-1
11284	PNKP	polynucleotide kinase 3'-phosphatase
8611	PPAP2A	phosphatidic acid phosphatase type 2A
8613	PPAP2B	phosphatidic acid phosphatase type 2B
8612	PPAP2C	phosphatidic acid phosphatase type 2C
5475	PPEF1	protein phosphatase, EF hand calcium-binding domain 1

5470	PPEF2	protein phosphatase, EF hand calcium-binding domain 2
8500	PPFIA1	protein tyrosine phosphatase, receptor type, f polypeptide (PTPRF),interacting protein (liprin), alpha 1
8499	PPFIA2	protein tyrosine phosphatase, receptor type, f polypeptide (PTPRF),interacting protein (liprin), alpha 2
8541	PPFIA3	protein tyrosine phosphatase, receptor type, f polypeptide (PTPRF),interacting protein (liprin), alpha 3
8497	PPFIA4	protein tyrosine phosphatase, receptor type, f polypeptide (PTPRF),interacting protein (liprin), alpha 4
5494	PPM1A	protein phosphatase 1A (formerly 2C), magnesium-dependent, alpha isoform
5495	PPM1B	protein phosphatase 1B (formerly 2C), magnesium-dependent, beta isoform
8493	PPM1D	protein phosphatase 1D magnesium-dependent, delta isoform
22843	PPM1E	protein phosphatase 1E (PP2C domain containing)
9647	PPM1F	protein phosphatase 1F (PP2C domain containing)
5496	PPM1G	protein phosphatase 1G (formerly 2C), magnesium-dependent, gamma isoform
152926	PPM1K	protein phosphatase 1K (PP2C domain containing)
151742	PPM1L	protein phosphatase 1 (formerly 2C)-like
132160	PPM1M	protein phosphatase 1M (PP2C domain containing)
54704	PPM2C	protein phosphatase 2C, magnesium-dependent, catalytic subunit
5500	PPP1CB	protein phosphatase 1, catalytic subunit, beta isoform
5501	PPP1CC	protein phosphatase 1, catalytic subunit, gamma isoform
5514	PPP1R10	protein phosphatase 1, regulatory subunit 10
6992	PPP1R11	protein phosphatase 1, regulatory (inhibitor) subunit 11
4659	PPP1R12A	protein phosphatase 1, regulatory (inhibitor) subunit 12A
4660	PPP1R12B	protein phosphatase 1, regulatory (inhibitor) subunit 12B
54776	PPP1R12C	protein phosphatase 1, regulatory (inhibitor) subunit 12C
23368	PPP1R13B	protein phosphatase 1, regulatory (inhibitor) subunit 13B
94274	PPP1R14A	protein phosphatase 1, regulatory (inhibitor) subunit 14A
26472	PPP1R14B	protein phosphatase 1, regulatory (inhibitor) subunit 14B
81706	PPP1R14C	protein phosphatase 1, regulatory (inhibitor) subunit 14C
54866	PPP1R14D	protein phosphatase 1, regulatory (inhibitor) subunit 14D
23645	PPP1R15A	protein phosphatase 1, regulatory (inhibitor) subunit 15A
84919	PPP1R15B	protein phosphatase 1, regulatory (inhibitor) subunit 15B
84988	PPP1R16A	protein phosphatase 1, regulatory (inhibitor) subunit 16A
26051	PPP1R16B	protein phosphatase 1, regulatory (inhibitor) subunit 16B
5502	PPP1R1A	protein phosphatase 1, regulatory (inhibitor) subunit 1A
84152	PPP1R1B	protein phosphatase 1, regulatory (inhibitor) subunit 1B (dopamineand cAMP regulated phosphoprotein, DARPP-32)
151242	PPP1R1C	protein phosphatase 1, regulatory (inhibitor) subunit 1C
5504	PPP1R2	protein phosphatase 1, regulatory (inhibitor) subunit 2
5506	PPP1R3A	protein phosphatase 1, regulatory (inhibitor) subunit 3A (glycogen and sarcoplasmic reticulum binding subunit, skeletal [illegible]
79660	PPP1R3B	protein phosphatase 1, regulatory (inhibitor) subunit 3B
5507	PPP1R3C	protein phosphatase 1, regulatory (inhibitor) subunit 3C
5509	PPP1R3D	protein phosphatase 1, regulatory subunit 3D
5510	PPP1R7	protein phosphatase 1, regulatory subunit 7
5511	PPP1R8	protein phosphatase 1, regulatory (inhibitor) subunit 8
55607	PPP1R9A	protein phosphatase 1, regulatory (inhibitor) subunit 9A
84687	PPP1R9B	protein phosphatase 1, regulatory subunit 9B, spinophilin
5515	PPP2CA	protein phosphatase 2 (formerly 2A), catalytic subunit, alpha isoform
5516	PPP2CB	protein phosphatase 2 (formerly 2A), catalytic subunit, beta isoform
5518	PPP2R1A	protein phosphatase 2 (formerly 2A), regulatory subunit A (PR 65), alpha isoform
5519	PPP2R1B	protein phosphatase 2 (formerly 2A), regulatory subunit A (PR 65), beta isoform
5520	PPP2R2A	protein phosphatase 2 (formerly 2A), regulatory subunit B (PR 52), alpha isoform
5521	PPP2R2B	protein phosphatase 2 (formerly 2A), regulatory subunit B (PR 52), beta isoform
5522	PPP2R2C	protein phosphatase 2 (formerly 2A), regulatory subunit B (PR 52), gamma isoform
55844	PPP2R2D	protein phosphatase 2, regulatory subunit B, delta isoform
5523	PPP2R3A	protein phosphatase 2 (formerly 2A), regulatory subunit B'', alpha
28227	PPP2R3B	protein phosphatase 2 (formerly 2A), regulatory subunit B'', beta
5524	PPP2R4	protein phosphatase 2A, regulatory subunit B' (PR 53)
5525	PPP2R5A	protein phosphatase 2, regulatory subunit B (B56), alpha isoform
5526	PPP2R5B	protein phosphatase 2, regulatory subunit B (B56), beta isoform
5527	PPP2R5C	protein phosphatase 2, regulatory subunit B (B56), gamma isoform
5528	PPP2R5D	protein phosphatase 2, regulatory subunit B (B56), delta isoform
5529	PPP2R5E	protein phosphatase 2, regulatory subunit B (B56), epsilon isoform
5530	PPP3CA	protein phosphatase 3 (formerly 2B), catalytic subunit, alpha isoform (calcineurin A alpha)
5532	PPP3CB	protein phosphatase 3 (formerly 2B), catalytic subunit, beta isoform (calcineurin A beta)
5533	PPP3CC	protein phosphatase 3 (formerly 2B), catalytic subunit, gamma isoform (calcineurin A gamma)
5534	PPP3R1	protein phosphatase 3 (formerly 2B), regulatory subunit B, 19kDa, alpha isoform (calcineurin B, type I)
5531	PPP4C	protein phosphatase 4 (formerly X), catalytic subunit
9989	PPP4R1	protein phosphatase 4, regulatory subunit 1
55370	PPP4R1L	protein phosphatase 4, regulatory subunit 1-like
5536	PPP5C	protein phosphatase 5, catalytic subunit
5537	PPP6C	protein phosphatase 6, catalytic subunit
5723	PSPH	phosphoserine phosphatase
5728	PTEN	phosphatase and tensin homolog (mutated in multiple advanced cancers 1)
7803	PTP4A1	protein tyrosine phosphatase type IVA, member 1
8073	PTP4A2	protein tyrosine phosphatase type IVA, member 2
11156	PTP4A3	protein tyrosine phosphatase type IVA, member 3
138639	PTPDC1	protein tyrosine phosphatase domain containing 1
9200	PTPLA	protein tyrosine phosphatase-like (proline instead of catalytic arginine), member a
114971	PTPMT1	protein tyrosine phosphatase, mitochondrial 1
5770	PTPN1	protein tyrosine phosphatase, non-receptor type 1
5781	PTPN11	protein tyrosine phosphatase, non-receptor type 11 (Noonan syndrome1)
5782	PTPN12	protein tyrosine phosphatase, non-receptor type 12
5783	PTPN13	protein tyrosine phosphatase, non-receptor type 13 (APO-1/CD95(Fas)-associated phosphatase)
5784	PTPN14	protein tyrosine phosphatase, non-receptor type 14
26469	PTPN18	protein tyrosine phosphatase, non-receptor type 18 (brain-derived)
5771	PTPN2	protein tyrosine phosphatase, non-receptor type 2

26095	PTPN20	protein tyrosine phosphatase, non-receptor type 20
11099	PTPN21	protein tyrosine phosphatase, non-receptor type 21
26191	PTPN22	protein tyrosine phosphatase, non-receptor type 22 (lymphoid)
25930	PTPN23	protein tyrosine phosphatase, non-receptor type 23
5774	PTPN3	protein tyrosine phosphatase, non-receptor type 3
5775	PTPN4	protein tyrosine phosphatase, non-receptor type 4 (megakaryocyte)
84867	PTPN5	protein tyrosine phosphatase, non-receptor type 5(striatum-enriched)
5777	PTPN6	protein tyrosine phosphatase, non-receptor type 6
5778	PTPN7	protein tyrosine phosphatase, non-receptor type 7
5780	PTPN9	protein tyrosine phosphatase, non-receptor type 9
140885	PTPNS1	protein tyrosine phosphatase, non-receptor type substrate 1
128646	PTPNS1L2	protein tyrosine phosphatase, non-receptor type substrate 1-like 2
284759	PTPNS1L3	protein tyrosine phosphatase, non-receptor type substrate 1-like 3
5786	PTPRA	protein tyrosine phosphatase, receptor type, A
5787	PTPRB	protein tyrosine phosphatase, receptor type, B
5788	PTPRC	protein tyrosine phosphatase, receptor type, C
5790	PTPRCAP	protein tyrosine phosphatase, receptor type, C-associated protein
5789	PTPRD	protein tyrosine phosphatase, receptor type, D
5791	PTPRE	protein tyrosine phosphatase, receptor type, E
5792	PTPRF	protein tyrosine phosphatase, receptor type, F
5793	PTPRG	protein tyrosine phosphatase, receptor type, G
5794	PTPRH	protein tyrosine phosphatase, receptor type, H
5795	PTPRJ	protein tyrosine phosphatase, receptor type, J
5796	PTPRK	protein tyrosine phosphatase, receptor type, K
5797	PTPRM	protein tyrosine phosphatase, receptor type, M
5798	PTPRN	protein tyrosine phosphatase, receptor type, N
5799	PTPRN2	protein tyrosine phosphatase, receptor type, N polypeptide 2
5800	PTPRO	protein tyrosine phosphatase, receptor type, O
374462	PTPRQ	protein tyrosine phosphatase, receptor type, Q
5801	PTPRR	protein tyrosine phosphatase, receptor type, R
5802	PTPRS	protein tyrosine phosphatase, receptor type, S
11122	PTPRT	protein tyrosine phosphatase, receptor type, T
10076	PTPRU	protein tyrosine phosphatase, receptor type, U
5803	PTPRZ1	protein tyrosine phosphatase, receptor-type, Z polypeptide 1
8732	RNGTT	RNA guanylyltransferase and 5'-phosphatase
6305	SBF1	SET binding factor 1
81846	SBF2	SET binding factor 2
81537	SGPP1	sphingosine-1-phosphate phosphatase 1
51763	SKIP	skeletal muscle and kidney enriched inositol phosphatase
6815	STYX	serine/threonine/tyrosine interacting protein
51657	DUSP24	dual specificity phosphatase 24 (putative)
8867	SYNJ1	synaptojanin 1
8871	SYNJ2	synaptojanin 2
160760	TA	-PP2C T-cell activation protein phosphatase 2C
23371	TENC1	tensin like C1 domain containing phosphatase (tensin 2)
7179	TPTE	transmembrane phosphatase with tensin homology
93492	TPTE2	transmembrane phosphoinositide 3-phosphatase and tensin homolog 2

Supplementary File 2

symbol	Fiber-FC	Fiber-Pvalue	PMMA-FC	PMMA-Pvalue
PIK3CB	2	0.00001	1	1
CDK10	2	0.00001	1	1
IRAK3	2	0.00001	1	1
DDR1	2	0.00001	1	1
SYK	2	0.00001	2	0.00001
PRKCZ	2	0.00001	1	1
EPHA3	2	0.00001	1	1
PIK3C2B	2	0.00001	1	1
PANK2	2	0.00001	-2	0.00001
DGKE	2	0.00001	2	0.00001
SRPK1	2	0.00001	-2	0.00001
MKNK1	2	0.00001	1	1
DGKZ	2	0.00001	2	0.00001
PRKCE	2	0.00001	2	0.00001
DKFZp761P042	2	0.00001	-2	0.00001
CAMKK2	2	0.00001	1	1
TXK	2	0.00001	1	1
RYK	2	0.00001	2	0.00001
CDK9	2	0.00001	1	1
VRK1	2	0.00001	1	1
NEK4	2	0.00001	1	1
STK40	2	0.00001	1	1
LOC340156	2	0.00001	1	1
MATK	2	0.00001	1	1
BRSK2	2	0.00001	1	1
PRKCB1	2	0.00001	1	1
RNASEL	2	0.00001	2	0.00001
CAMK2D	2	0.00001	1	1
EPHB2	2	0.00001	-2	0.00001
ROS1	2	0.00001	2	0.00001
PIP5K2B	2	0.00001	1	1
CDK8	2	0.00001	-2	0.00001
TTBK2	2	0.00001	2	0.00001
TK2	2	0.00001	-2	0.00001
MAP3K8	2	0.00001	2	0.00001
ALS2CR2	2	0.00001	2	0.00001
VRK3	2	0.00001	2	0.00001
LYN	2	0.00001	-2	0.00001
PGK1	2	0.00001	1	1
SCAP1	2	0.00001	1	1
BLK	2	0.00001	1	1
ULK1	2	0.00001	1	1

DCAMKL2	2	0.00001	-2	0.00001
ATM	2	0.00001	2	0.00001
MAP3K9	2	0.00001	1	1
NEK1	2	0.00001	2	0.00001
ICK	2	0.00001	1	1
CHUK	2	0.00001	-2	0.00001
MAP3K14	2	0.00001	1	1
ERBB3	2	0.00001	1	1
TNIK	2	0.00001	-2	0.00001
EPHB1	2	0.00001	2	0.00001
ITPKC	2	0.00001	-2	0.00001
HIPK4	2	0.00001	2	0.00001
LTK	2	0.00001	1	1
PSKH2	2	0.00001	1	1
TAOK2	2	0.00001	1	1
IKBKB	2	0.00001	1	1
LCK	2	0.00001	-2	0.00001
PRKCQ	2	0.00001	-2	0.00001
PCTK2	2	0.00001	1	1
PIK4CA	2	0.00001	1	1
MET	2	0.00001	1	1
PRKCI	2	0.00001	1	1
ARAF	2	0.00001	2	0.00001
PRKD1	2	0.00001	1	1
GRK7	2	0.00001	1	1
PHKG1	2	0.00001	1	1
KIAA0999	2	0.00001	-2	0.00001
STK32B	2	0.00001	1	1
PFKFB4	2	0.00001	2	0.00001
TRIB2	2	0.00001	1	1
ALK	2	0.00001	1	1
MAPK8	2	0.00001	1	1
STK11	2	0.00001	1	1
MAP3K7	2	0.00001	1	1
GRK6	2	0.00001	1	1
RIPK3	2	0.00001	2	0.00001
PFKFB3	2	0.00001	1	1
STK19	2	0.00001	2	0.00001
JAK3	2	0.00001	1	1
PIK3C2G	2	0.00001	-2	0.00001
ROR2	2	0.00001	-2	0.00001
TSSK2	2	0.00001	1	1
YSK4	2	0.00001	2	0.00001

AKT1	2	0.00001	-2	0.00001
MERTK	2	0.00001	1	1
STK39	2	0.00001	-2	0.00001
FYN	2	0.00001	2	0.00001
FLT4	2	0.00001	1	1
ULK4	2	0.00001	1	1
TSSK4	2	0.00001	-2	0.00001
RET	2	0.00001	1	1
MAP3K12	2	0.00001	1	1
STK17B	2	0.00001	1	1
PRKX	2	0.00001	2	0.00001
CDK2	2	0.00001	1	1
MARK2	2	0.00001	1	1
ITK	2	0.00001	2	0.00001
MAST4	2	0.00001	1	1
YES1	2	0.00001	2	0.00001
PFKP	2	0.00001	1	1
KSR	2	0.00001	1	1
NEK5	2	0.00001	1	1
CDK5	2	0.00001	2	0.00001
MASK	2	0.00001	2	0.00001
ROCK1	2	0.00001	-2	0.00001
PAK4	2	0.00001	-2	0.00001
CDC2L6	2	0.00001	2	0.00001
EXOSC10	2	0.00001	1	1
MAP3K10	2	0.00001	1	1
TRPM7	2	0.00001	2	0.00001
WNK2	2	0.00001	2	0.00001
AMHR2	2	0.00001	1	1
FGFR4	2	0.00001	1	1
COASY	2	0.00001	2	0.00001
BTK	2	0.00001	2	0.00001
MARK3	2	0.00001	2	0.00001
DYRK4	2	0.00001	2	0.00001
MLCK	2	0.00001	1	1
RPS6KB2	2	0.00001	2	0.00001
EPHA4	2	0.00001	1	1
PTP4A1	2	0.00001	-2	0.00001
CDKN3	2	0.00001	1	1
PPFIA4	2	0.00001	-2	0.00001
PPP1R8	2	0.00001	1	1
PTPN5	2	0.00001	1	1
STYXL1	2	0.00001	1	1

SYNJ2	2	0.00001	-2	0.00001
SGPP1	2	0.00001	1	1
ACPL2	2	0.00001	2	0.00001
PTPN9	2	0.00001	1	1
PPM1E	2	0.00001	-2	0.00001
DUSP18	2	0.00001	-2	0.00001
PTPNS1L3	2	0.00001	1	1
ACPP	2	0.00001	1	1
PPP6C	2	0.00001	2	0.00001
PPP1R1A	2	0.00001	1	1
FBP2	2	0.00001	1	1
PTPN13	2	0.00001	2	0.00001
LHPP	2	0.00001	1	1
DUSP15	2	0.00001	1	1
PTPMT1	2	0.00001	1	1
PFKFB1	2	0.00001	-2	0.00001
PTPN1	2	0.00001	-2	0.00001
INPP4B	2	0.00001	-2	0.00001
PPP3CA	2	0.00001	1	1
PPP2R4	2	0.00001	1	1
PPP2R1B	2	0.00001	2	0.00001
TPTE2	2	0.00001	1	1
PPP1R14C	2	0.00001	1	1
CDC25C	2	0.00001	2	0.00001
DUSP5	2	0.00001	2	0.00001
ENPP3	2	0.00001	1	1
PPM1A	2	0.00001	2	0.00001
PPP1R3A	2	0.00001	1	1
PPP2R5A	2	0.00001	2	0.00001
DUSP21	2	0.00001	1	1
CDC25B	2	0.00001	2	0.00001
DUSP2	2	0.00001	1	1
TA	2	0.00001	-2	0.00001
RNGTT	2	0.00001	2	0.00001
PPEF2	2	0.00001	1	1
PTPDC1	2	0.00001	2	0.00001
PTPN14	2	0.00001	1	1
DUSP13	2	0.00001	1	1
PPM1M	2	0.00001	1	1
ATR	-2	0.00001	1	1
CALM2	-2	0.00001	1	1
DAPK2	-2	0.00001	2	0.00001
PCTK3	-2	0.00001	1	1

MAP4K1	-2	0.00001	-2	0.00001
TRIM33	-2	0.00001	1	1
STK24	-2	0.00001	-2	0.00001
AURKC	-2	0.00001	1	1
EEF2K	-2	0.00001	-2	0.00001
SCYL1	-2	0.00001	1	1
MAPK4	-2	0.00001	1	1
TEC	-2	0.00001	1	1
PAK2	-2	0.00001	2	0.00001
TESK1	-2	0.00001	-2	0.00001
CSNK1A1	-2	0.00001	1	1
EIF2AK2	-2	0.00001	2	0.00001
BC007901	-2	0.00001	1	1
ALS2CR7	-2	0.00001	2	0.00001
STK16	-2	0.00001	-2	0.00001
PAK3	-2	0.00001	-2	0.00001
BRSK1	-2	0.00001	-2	0.00001
ETNK2	-2	0.00001	1	1
GRK4	-2	0.00001	-2	0.00001
TRIB1	-2	0.00001	1	1
FLJ32685	-2	0.00001	1	1
TGFBR2	-2	0.00001	1	1
CHEK1	-2	0.00001	1	1
NEK7	-2	0.00001	1	1
PRKG1	-2	0.00001	-2	0.00001
RIPK1	-2	0.00001	-2	0.00001
MAPK13	-2	0.00001	1	1
PDXK	-2	0.00001	-2	0.00001
CAMK1D	-2	0.00001	1	1
DGKD	-2	0.00001	2	0.00001
ILK	-2	0.00001	1	1
TJP2	-2	0.00001	-2	0.00001
PRKG2	-2	0.00001	2	0.00001
NEK3	-2	0.00001	1	1
NTRK1	-2	0.00001	2	0.00001
MAST3	-2	0.00001	-2	0.00001
PKM2	-2	0.00001	1	1
NME5	-2	0.00001	1	1
SGK2	-2	0.00001	1	1
PIK3R4	-2	0.00001	-2	0.00001
CDKL4	-2	0.00001	-2	0.00001
TRRAP	-2	0.00001	1	1
LYK5	-2	0.00001	-2	0.00001

CAMK4	-2	0.00001	1	1
PDK3	-2	0.00001	1	1
ALPK3	-2	0.00001	1	1
CLK4	-2	0.00001	2	0.00001
PDK4	-2	0.00001	1	1
MAP4K2	-2	0.00001	1	1
ADRBK2	-2	0.00001	1	1
TRIM28	-2	0.00001	-2	0.00001
PDIK1L	-2	0.00001	1	1
FN3K	-2	0.00001	1	1
PRKCH	-2	0.00001	-2	0.00001
MAPKAPK3	-2	0.00001	-2	0.00001
CDC42BPG	-2	0.00001	1	1
TEK	-2	0.00001	1	1
STK6	-2	0.00001	-2	0.00001
PDGFRA	-2	0.00001	1	1
NTRK2	-2	0.00001	1	1
MAP2K7	-2	0.00001	2	0.00001
PHKA2	-2	0.00001	1	1
PDGFRB	-2	0.00001	1	1
HIPK1	-2	0.00001	2	0.00001
ALPK2	-2	0.00001	1	1
LRRK2	-2	0.00001	1	1
LIMK2	-2	0.00001	-2	0.00001
MAP3K5	-2	0.00001	2	0.00001
NRK	-2	0.00001	1	1
ABL2	-2	0.00001	-2	0.00001
SCAP2	-2	0.00001	-2	0.00001
SGKL	-2	0.00001	-2	0.00001
PANK4	-2	0.00001	1	1
SBK1	-2	0.00001	1	1
KIAA2002	-2	0.00001	1	1
RIPK2	-2	0.00001	2	0.00001
ABL1	-2	0.00001	1	1
NADK	-2	0.00001	1	1
FES	-2	0.00001	-2	0.00001
EPHA2	-2	0.00001	1	1
STK38	-2	0.00001	1	1
GUCY2F	-2	0.00001	1	1
DDR2	-2	0.00001	1	1
ADCK2	-2	0.00001	2	0.00001
TNK2	-2	0.00001	1	1
MAPK14	-2	0.00001	2	0.00001

CSNK2A2	-2	0.00001	1	1
BMP2K	-2	0.00001	1	1
PACE-1	-2	0.00001	1	1
XYLB	-2	0.00001	-2	0.00001
RAGE	-2	0.00001	1	1
MLKL	-2	0.00001	1	1
PASK	-2	0.00001	1	1
MAPKAPK5	-2	0.00001	2	0.00001
CAMK2A	-2	0.00001	2	0.00001
AK1	-2	0.00001	1	1
MYO3A	-2	0.00001	1	1
PIP5K2C	-2	0.00001	-2	0.00001
CERK	-2	0.00001	1	1
DAPK1	-2	0.00001	2	0.00001
CLK3	-2	0.00001	1	1
TNNI3K	-2	0.00001	1	1
LMTK3	-2	0.00001	1	1
PTK7	-2	0.00001	1	1
CDK4	-2	0.00001	1	1
BRD3	-2	0.00001	-2	0.00001
DYRK2	-2	0.00001	1	1
CDKL1	-2	0.00001	2	0.00001
RPS6KA1	-2	0.00001	2	0.00001
FLT3	-2	0.00001	1	1
FGFR3	-2	0.00001	1	1
PKLR	-2	0.00001	1	1
LIMK1	-2	0.00001	1	1
FLJ25006	-2	0.00001	2	0.00001
BRD2	-2	0.00001	1	1
PIK3C2A	-2	0.00001	1	1
STK32C	-2	0.00001	1	1
SNF1LK2	-2	0.00001	-2	0.00001
PPP2R2C	-2	0.00001	1	1
PPP1R3C	-2	0.00001	1	1
PPP2R2A	-2	0.00001	1	1
PLEKHE1	-2	0.00001	1	1
DAPP1	-2	0.00001	1	1
PTPRB	-2	0.00001	1	1
DUSP11	-2	0.00001	-2	0.00001
PPM1D	-2	0.00001	1	1
PPP2R2B	-2	0.00001	-2	0.00001
DUSP8	-2	0.00001	1	1
ACP5	-2	0.00001	-2	0.00001

ALPL	-2	0.00001	1	1
ALPI	-2	0.00001	1	1
PPAP2B	-2	0.00001	1	1
DUSP3	-2	0.00001	1	1
PPP1R10	-2	0.00001	1	1
PPP4R1	-2	0.00001	1	1
PPAP2A	-2	0.00001	1	1
PTPRF	-2	0.00001	2	0.00001
CDC14C	-2	0.00001	1	1
PPP1R9A	-2	0.00001	1	1
CTDP1	-2	0.00001	1	1
CTDSP2	-2	0.00001	1	1
PPP2CB	-2	0.00001	1	1
PHOSPHO1	-2	0.00001	-2	0.00001
IMPA1	-2	0.00001	1	1
SBF2	-2	0.00001	1	1
PPM1B	-2	0.00001	1	1
PME	-2	0.00001	1	1
PTPN11	-2	0.00001	1	1
PPP1R12C	-2	0.00001	1	1
PTPN3	-2	0.00001	2	0.00001
EYA3	-2	0.00001	2	0.00001
EYA4	-2	0.00001	-2	0.00001
PPP2R5B	-2	0.00001	1	1
PPM1G	-2	0.00001	-2	0.00001
MTMR7	-2	0.00001	-2	0.00001
PTPRU	-2	0.00001	-2	0.00001
SYNJ1	-2	0.00001	2	0.00001
PTPRR	-2	0.00001	1	1
SKIP	-2	0.00001	1	1
PPM1L	-2	0.00001	2	0.00001
PHLPPL	-2	0.00001	1	1
ENPP2	-2	0.00001	-2	0.00001
ACP2	-2	0.00001	1	1
FBP1	-2	0.00001	2	0.00001
TAF1	1	1	2	0.00001
DMPK	1	1	2	0.00001
TAOK1	1	1	2	0.00001
EIF2AK1	1	1	2	0.00001
RPS6KB1	1	1	2	0.00001
DGKI	1	1	2	0.00001
MASTL	1	1	2	0.00001
SMG1	1	1	2	0.00001

UHMK1	1	1	2	0.00001
RPS6KA6	1	1	2	0.00001
WNK4	1	1	2	0.00001
CLK1	1	1	2	0.00001
NTRK3	1	1	2	0.00001
STK3	1	1	2	0.00001
TNK1	1	1	2	0.00001
MYO3B	1	1	2	0.00001
MINK1	1	1	2	0.00001
PRKACG	1	1	2	0.00001
NLK	1	1	2	0.00001
ROCK2	1	1	2	0.00001
MKNK2	1	1	2	0.00001
CDK6	1	1	2	0.00001
GSG2	1	1	2	0.00001
MPP2	1	1	2	0.00001
PCTK1	1	1	2	0.00001
AK3L1	1	1	2	0.00001
LATS1	1	1	2	0.00001
TLK1	1	1	2	0.00001
SRPK2	1	1	2	0.00001
FGFRL1	1	1	2	0.00001
BRDT	1	1	2	0.00001
MAST1	1	1	2	0.00001
GCK	1	1	2	0.00001
PIP5K1B	1	1	2	0.00001
FRK	1	1	2	0.00001
FGFR2	1	1	2	0.00001
DYRK1B	1	1	2	0.00001
MAP3K15	1	1	2	0.00001
AKT3	1	1	2	0.00001
FUK	1	1	2	0.00001
NRBP2	1	1	2	0.00001
PRKY	1	1	2	0.00001
STK17A	1	1	2	0.00001
ULK2	1	1	2	0.00001
EPHA8	1	1	2	0.00001
HIPK3	1	1	2	0.00001
PIM2	1	1	2	0.00001
GALK2	1	1	2	0.00001
MPP3	1	1	2	0.00001
MAPK9	1	1	2	0.00001
MAP2K2	1	1	2	0.00001

CABC1	1	1	2	0.00001
AK5	1	1	2	0.00001
PHKB	1	1	2	0.00001
PXK	1	1	2	0.00001
MAP2K4	1	1	2	0.00001
DGKH	1	1	2	0.00001
PTPN7	1	1	2	0.00001
PPP2R3A	1	1	2	0.00001
PPM2C	1	1	2	0.00001
PTP4A3	1	1	2	0.00001
PPEF1	1	1	2	0.00001
DUSP12	1	1	2	0.00001
INPP1	1	1	2	0.00001
PTPRT	1	1	2	0.00001
KIAA1274	1	1	2	0.00001
INPP5E	1	1	2	0.00001
MTMR2	1	1	2	0.00001
PTPRS	1	1	2	0.00001
PTPN22	1	1	2	0.00001
EPM2A	1	1	2	0.00001
PSPH	1	1	2	0.00001
DUSP1	1	1	2	0.00001
PPP4C	1	1	2	0.00001
TENC1	1	1	2	0.00001
PTPN6	1	1	2	0.00001
DNAJC6	1	1	2	0.00001
CILP	1	1	2	0.00001
DUSP22	1	1	2	0.00001
PPP5C	1	1	2	0.00001
PRKAA1	1	1	-2	0.00001
PMVK	1	1	-2	0.00001
COL4A3BP	1	1	-2	0.00001
TPK1	1	1	-2	0.00001
INSR	1	1	-2	0.00001
CAMK1	1	1	-2	0.00001
ZAP70	1	1	-2	0.00001
PLK4	1	1	-2	0.00001
IGF1R	1	1	-2	0.00001
CSK	1	1	-2	0.00001
FGR	1	1	-2	0.00001
HIPK2	1	1	-2	0.00001
MELK	1	1	-2	0.00001
OXSR1	1	1	-2	0.00001

MOS	1	1	-2	0.00001
TTK	1	1	-2	0.00001
MAP2K1	1	1	-2	0.00001
PAK1	1	1	-2	0.00001
BUB1B	1	1	-2	0.00001
RPS6KA3	1	1	-2	0.00001
ITPKB	1	1	-2	0.00001
GUCY2C	1	1	-2	0.00001
CKM	1	1	-2	0.00001
GSK3A	1	1	-2	0.00001
BUB1	1	1	-2	0.00001
GUCY2D	1	1	-2	0.00001
PANK3	1	1	-2	0.00001
TTN	1	1	-2	0.00001
VRK2	1	1	-2	0.00001
FLJ23356	1	1	-2	0.00001
STK23	1	1	-2	0.00001
IRAK4	1	1	-2	0.00001
PINK1	1	1	-2	0.00001
SPHK1	1	1	-2	0.00001
AK3	1	1	-2	0.00001
PIP5K3	1	1	-2	0.00001
FRAP1	1	1	-2	0.00001
ADCK5	1	1	-2	0.00001
CLK2	1	1	-2	0.00001
APEG1	1	1	-2	0.00001
SNF1LK	1	1	-2	0.00001
GNE	1	1	-2	0.00001
CDK3	1	1	-2	0.00001
NPR1	1	1	-2	0.00001
DLG3	1	1	-2	0.00001
CDC2L5	1	1	-2	0.00001
TEX14	1	1	-2	0.00001
ERN1	1	1	-2	0.00001
RPS6KA4	1	1	-2	0.00001
PLK1	1	1	-2	0.00001
RIOK1	1	1	-2	0.00001
PRPF4B	1	1	-2	0.00001
PRKACB	1	1	-2	0.00001
PFKFB2	1	1	-2	0.00001
GRK1	1	1	-2	0.00001
MAP3K13	1	1	-2	0.00001
PIK3CA	1	1	-2	0.00001

KALRN	1	1	-2	0.00001
ADCK4	1	1	-2	0.00001
ALPPL2	1	1	-2	0.00001
CAMK2G	1	1	-2	0.00001
CDC14B	1	1	-2	0.00001
MTMR3	1	1	-2	0.00001
ACP6	1	1	-2	0.00001
PTPN18	1	1	-2	0.00001
DUSP19	1	1	-2	0.00001
MTM1	1	1	-2	0.00001
PPP1R1B	1	1	-2	0.00001
PPFIA3	1	1	-2	0.00001
DUPD1	1	1	-2	0.00001
PPP1R7	1	1	-2	0.00001
CDC25A	1	1	-2	0.00001
PTPRE	1	1	-2	0.00001
PDP2	1	1	-2	0.00001
PPP2R5E	1	1	-2	0.00001
PTPN2	1	1	-2	0.00001
PTPRN2	1	1	-2	0.00001
PTPRN	1	1	-2	0.00001
PTP4A2	1	1	-2	0.00001
PPP3R1	1	1	-2	0.00001
PTPRD	1	1	-2	0.00001
DUSP6	1	1	-2	0.00001
